# Long-Term Programming of Antigen-Specific Immunity from Gene Expression Signatures in the PBMC of Rhesus Macaques Immunized with an SIV DNA Vaccine

**DOI:** 10.1371/journal.pone.0019681

**Published:** 2011-06-20

**Authors:** Sarah E. Belisle, Jiangmei Yin, Devon J. Shedlock, Anlan Dai, Jian Yan, Lauren Hirao, Michele A. Kutzler, Mark G. Lewis, Hanne Andersen, Simon M. Lank, Julie A. Karl, David H. O'Connor, Amir Khan, Niranjan Sardesai, Jean Chang, Lauri Aicher, Robert E. Palermo, David B. Weiner, Michael G. Katze, Jean Boyer

**Affiliations:** 1 Department of Microbiology, University of Washington, Seattle, Washington, United States of America; 2 Department of Pathology and Laboratory Medicine, University of Pennsylvania School of Medicine, Philadelphia, Pennsylvania, United States of America; 3 Department of Medicine, Drexel University College of Medicine, Philadelphia, Pennsylvania, United States of America; 4 Research Section, Bioqual, Rockville, Maryland, United States of America; 5 Wisconsin National Primate Research Center, University of Wisconsin-Madison, Madison, Wisconsin, United States of America; 6 Inovio Pharmaceuticals, Blue Bell, Pennsylvania, United States of America; Tulane University, United States of America

## Abstract

While HIV-1-specific cellular immunity is thought to be critical for the suppression of viral replication, the correlates of protection have not yet been determined. Rhesus macaques (RM) are an important animal model for the study and development of vaccines against HIV/AIDS. Our laboratory has helped to develop and study DNA-based vaccines in which recent technological advances, including genetic optimization and *in vivo* electroporation (EP), have helped to dramatically boost their immunogenicity. In this study, RMs were immunized with a DNA vaccine including individual plasmids encoding SIV *gag*, *env*, and *pol* alone, or in combination with a molecular adjuvant, plasmid DNA expressing the chemokine ligand 5 (RANTES), followed by EP. Along with standard immunological assays, flow-based activation analysis without *ex vivo* restimulation and high-throughput gene expression analysis was performed. Strong cellular immunity was induced by vaccination which was supported by all assays including PBMC microarray analysis that identified the up-regulation of 563 gene sequences including those involved in interferon signaling. Furthermore, 699 gene sequences were differentially regulated in these groups at peak viremia following SIVmac251 challenge. We observed that the RANTES-adjuvanted animals were significantly better at suppressing viral replication during chronic infection and exhibited a distinct pattern of gene expression which included immune cell-trafficking and cell cycle genes. Furthermore, a greater percentage of vaccine-induced central memory CD8+ T-cells capable of an activated phenotype were detected in these animals as measured by activation analysis. Thus, co-immunization with the RANTES molecular adjuvant followed by EP led to the generation of cellular immunity that was transcriptionally distinct and had a greater protective efficacy than its DNA alone counterpart. Furthermore, activation analysis and high-throughput gene expression data may provide better insight into mechanisms of viral control than may be observed using standard immunological assays.

## Introduction

The development of an effective HIV vaccine has proven to be a significant challenge and the exact nature of a protective immune profile remains elusive. Importantly, the RV144 trial is the first study that has demonstrated any sort of efficacy [1]. It was a randomized trial of the “prime-boost” combination of ALVAC HIV (prime) and AIDSVAX B/E (boost) versus placebo conducted in more than 16,000 HIV-negative volunteers in Thailand. A relative reduction in viral infection (31.1%) was observed in vaccinated subjects with an absolute difference of 0·28%. Furthermore, binding Abs were observed in most vaccinated subjects suggesting their importance in protection from infection [1]. However, the Ab response alone is not likely the only contributor to efficacy and a number of studies have indicated that a cellular immunity also contributes to protection against HIV infection in humans and SIV infection in RMs [2], [3]; high CD4^+^ T-cell and CD8^+^ T-cell gamma interferon (IFN- γ) responses are associated with better viral control during chronic SIV infection [4]. In human studies, the CD8^+^ T-cell response and the HIV-1 viral load during primary infection have been shown to be inversely related [5], [6], [7], [8], [9], [10]. Studies of chronic infection suggest that non-progressors maintain HIV-specific CD8^+^ T-cell proliferation [11] and polyfunctional HIV-specific CD8^+^ T-cell responses [12]. Finally, *in vivo* depletion of CD8^+^ T-cells in SIV- or SHIV89.6p-infected RMs implicates the importance of T-cells in the control of viral replication during both acute [13] and chronic [14] infection. More recently, impairment of CD4^+^ and CD8^+^ cell function through the up-regulation of inhibitory T-cell surface markers has been observed with disease progression during HIV-1 and SIV infection [15], [16], [17], [18], [19]. One of the goals of the current study was to develop an SIV DNA vaccine that induces strong SIV-specific cellular immune responses by optimizing its delivery using *in vivo* electroporation (EP) and its expression using co-delivery of a plasmid molecular adjuvant encoding the chemokine ligand 5 (RANTES).

RANTES (or Regulated upon Activation, Normal T-cell Expressed, and Secreted), member of the CC-chemokine family and similar to other CCR5 ligand-macrophage inflammatory proteins 1a (MIP-1a) and 1b (MIP-1b), can inhibit the entry of HIV type 1 (HIV-1) strains that use CCR5 as an entry co-receptor with CD4 (R5 strains) [20], [21]. These chemokines inhibit entry of the virus at binding sites located on the same receptor, leading to suppression of CCR5-tropic (R5-tropic) HIV-1 infections [22]. In addition, RANTES has been demonstrated to be predominantly associated with T helper type-1 (Th1) responses [23], [24], [25]. An immune response polarized towards a Th1 phenotype is associated with a reduced viral load and non-progression of disease during HIV-1 infection [26]. Furthermore, RANTES has been found to enhance cellular immune responses resulting in a more effective immune-modulating effect against HIV-1-related viruses in rodent and monkey models [27], [28], [29], [30]. Thus, based on these previous studies describing its immunogenicity in relation to HIV, RANTES was selected as a molecular adjuvant in the present study.

Since standard immunological assays measuring the magnitude and functional capacity of vaccine-specific T cell responses has provided little correlation with protection from HIV disease [31], we used several non-standard assays to ascertain possible mechanisms of viral suppression. Firstly, we employed flow-based activation analysis in the absence of *ex vivo* restimulation which has been shown to correlate T cell activation with vaccine-induced immune responses and is highly specific with minimal bystander effects [32]. Next, to examine vaccine-induced genetic signatures we used microarrays which have provided during both HIV-1 and SIV infection numerous mechanistic insights into pathogenesis including the timing of innate gene expression, interferon stimulated genes, the regulation of apoptosis in different cell types, and many others [33], [34], [35], [36]. However, the application of high-throughput global gene expression analysis to HIV and SIV vaccine studies has generally been limited in number and these studies did not specifically examine expression in RM suppressors of SIV replication. Thus, the acquisition of high-throughput data in HIV and SIV vaccine studies will allow for sensitive interrogation of adaptive immune pathways previously theorized to influence efficacy and provide information about responses, such as innate responses, that have not been as well-characterized with regard to vaccine efficacy.

We demonstrate herein that a SIV DNA vaccine can induce strong cellular immunity and suppress SIV viral replication when administered in conjunction with a plasmid molecular adjuvant expressing RANTES. Vaccine-specific immune responses were assessed using standard immunological assays including both ELISpot and flow cytometry, but no direct correlation between immunity and viral load was found. We therefore employed non-standard immunological techniques including activation analysis in the absence of *ex vivo* restimulation [32] and genetic analysis to better analyze the data. While DNA vaccination alone induced a greater amount of SIV-specific effectors as measured by standard immunological assays, DNA vaccination adjuvanted by RANTES as measured by activation analysis generated a greater percentage of central memory CD8+ T-cells capable of an activated phenotype following the final immunization. Microarrays were used to examine the expression of 20,217 gene sequences in PBMCs from vaccinated animals that were stimulated *in vitro* with SIVpol peptides; 563 gene sequences distinguished the vaccine groups at eight months following immunization and 699 gene sequences distinguished them post-challenge at peak viremia. We found that vaccination was associated with an enhanced Th1 gene expression response including interferon signaling and chemokine response to SIVpol at both pre- and post- challenge, which was consistent with the standard immunological assays. Furthermore, that vaccination caused an up-regulation in the transcription of immune response pathways that included cytolysis-related genes at 8 months post-vaccination. Interestingly, we observed that a decrease in cell death-related genes correlated with vaccination at peak viremia. Lastly, a unique signature of genes whose expression distinguished the RANTES group was observed. These RANTES-specific gene expression patterns may suggest a long-term reprogramming of the immune response to SIVpol that is unique to the animals that received this adjuvant and could offer insight into the enhanced SIV suppression observed post-challenge.

## Materials and Methods

### Animals and infection

Rhesus macaques (Macaca mulatta) were housed at BIOQUAL, Inc. (Rockville, MD), in accordance with the standards of the American Association for Accreditation of Laboratory Animal Care. The protocol was approved by the BIOQUAL's Institutional Animal care and Use Committee under OLAW Assurance Number of A-3086-01. Bioqual is IAAALAC accredited and procedures were in accordance with the recommendations of the Weatherall report. The University of Pennsylvania Institutional Animal Care and Use Committee (IACUC) reviewed and approved all procedures carried out by BIOQUAL. Animals were allowed to acclimate for at least 30 days in quarantine prior to any experimentation. All animals received environmental enrichment throughout this study. All experimental procedures were performed under ketamine anesthesia and all efforts were made to minimize pain and suffering.

Animals were challenged eight months following the fourth immunization with 25 monkey infectious doses (MID) of SIVmac251 by the intrarectal route. Viral stocks were provided by Advanced BioSciences Laboratories, Inc (ABL Inc.; Kensington, MD) and titered by BIOQUAL, Inc. Plasma viral load was determined by quantitative Nucleic Acid Sequence Based Amplification (NASBA) assay performed by Advanced BioSciences Laboratories, Inc (ABL, Inc.; Kensington, MD) as previously described [37]. CD4+ T-cell numbers were also measured by ABL and titered by Bioqual Inc. (Rockville, MD).

### DNA plasmids and immunization

The DNA plasmids used in this study expressed the modified RM proteins for SIV gag (pSIVgag), pol (pSIVpol), or env (pSIVenv). Consensus sequences were generated with several modifications as follows. Expression was enhanced using a human IgE leader peptide [38], SIV env V1 and V2 regions were shortened by removing N-linked glycosylation sites, and the cytoplasmic tail was truncated to prevent envelope recycling. For SIV pol, three mutations were introduced to deactivate the protease, reverse transcriptase, and RNase H. The resulting optimized SIV DNA immunogens were codon- and RNA-optimized, synthesized, and cloned into the pVAX1 expression vector to create optimized expression constructs. Constructs were amplified and purified by Aldevron (Fargo, ND) and then formulated in 0.15 M citrate buffer of pH 6.7 with 0.25% bupivicaine in water.

The DNA alone group consisted of six RMs that were immunized at weeks 0, 8, 12 and 24 with a mixture of 1.5 mg each of pSIVgag, pSIVpol, and pSIVenv. The RANTES-adjuvanted group consisted of six RMs that received a mixture of 1.5 mg each of SIVgag, SIVenv, SIVpol and RANTES at weeks 0, 8, 12 and for the 4th immunization at week 24, received only DNA but no RANTES adjuvant. Lastly, a third group of six RMs were used as the negative control group (Naïve) and immunized with saline (Table 1). For each immunization, plasmid DNA was delivered into a single injection site in the quadriceps muscle followed by *in vivo* EP. All EP procedures were performed using the CELLECTRA™ device (Inovio Biomedical Corporation, The Woodlands, TX) at a constant current consisting of 3 pulses at 0.5 Amps, with each pulse being 52 ms in length and separated by 1 second intervals. This software-controlled device was designed to measure the tissue resistance immediately prior to plasmid delivery and generation of constant current square wave pulses, thus eliminating the risk of delivery outside the muscle tissue and potential plasmid loss [39], [40].

**Table 1:** Immunization schedule.

Immunization	Control group	DNA group	DNA+RANTES group
Week 0	Control	SIV gag	SIV gag
		SIV env	SIV env+RANTES
		SIV pol	SIV pol
Week 8	Control	SIV gag	SIV gag
		SIV env	SIV env+RANTES
		SIV pol	SIV pol
Week 12	Control	SIV gag	SIV gag
		SIV env	SIV env+RANTES
		SIV pol	SIV pol
Week 20	Control	SIV gag	SIV gag
		SIV env	SIV env
		SIV pol	SIV pol

### PBMC isolation

For the isolation of PBMCs, blood was collected in EDTA tubes, shipped overnight, transferred to conical tubes, and centrifuged at 2,000 rpm to pellet cells. Plasma was separated from cells and replaced with an equivalent volume of Hanks' Balanced Salt Solution (HBSS; Invitrogen, Grand Island, NY). PBMC were isolated by standard Ficoll-hypaque centrifugation and residual red blood cells were lysed using ammonium chloride-potassium (ACK) Lysing Buffer (Lonza, Walkersville, MD). Cells were washed twice with HBSS and then resuspended in complete culture medium (RPMI 1640 with 2 mM/L L-glutamine, 100 IU/ml each of penicillin and streptomycin, 55 µm/L β-mercaptoethanol, and 10% heat-inactivated fetal bovine serum (FBS)). Viable cells were counted using a 0.4% (w/v) Trypan Blue Solution (Mediatech Inc., Herndon, VA).

### IFN-γ ELISpot assay

RM ELISpot was performed as previously described [41]. Briefly, ELISpot assays using IFN-γ reagents (MabTech, Sweden) and nitrocellulose plates (Millipore, Billerica, MA) were performed according to the manufacturer's instructions. Ninety-six well MultiScreen-IP plates with hydrophobic PVDF filters (0.45 µm) (Millipore, Billerica, MA) were coated with IFN-γ capture antibody and incubated overnight at 4°C. After washing with sterile PBS, the wells were blocked with complete culture medium for at least 30 min at room temperature. The SIV peptide pools (15-mers overlapping by 11 amino acids; NIH AIDS Research & Reference Reagent Program, Germantown, MD) were diluted 1∶200 in culture medium and 100 µl was transferred into triplicate wells. Peptide matrices were made as previously described [42]. Cells in culture media were added to the wells at a concentration of 200,000 cells per well and the plate was incubated for 18–24 h at 37°C, 5% CO_2_. After washing with sterile PBS, biotinylated antibody (1 µg/ml) in PBS with 0.5% FBS was added and incubated for 2 h at room temperature. Wells were washed with sterile PBS and streptavidin-ALP was added at a concentration of 1 ng/ml in PBS with 0.5% PCS and incubated for 1 h at room temperature. Plates were washed again with sterile PBS and BCIP/NBT (Sigma, St. Louis, MO) was added and incubated until spots appeared. Reactions were stopped using tap water and plates were left overnight at room temperature to dry. A positive response was defined as greater than 50 spot forming cells (SFC) per million PBMCs and two times above background and enumerated using an ImmunoSpot® Analyzer and Immunospot® Version 3.0 software (Cellular Technology Ltd., Shaker Heights, OH), in the negative control wells from the wells containing peptides.

### T-cell proliferation and flow cytometry

Fresh PBMCs were incubated with CFSE (5 µM) for 8 min at 37°C. Cells were washed and incubated with medium alone (negative control), peptides (SIV239gag peptide mix, SIV239env peptide mix, SIV239pol peptide mix) at a concentration of 5 ug/mL, or Concanavalin A (ConA; positive control) for 5 days at 37°C in 96-well plates. Cultures with medium alone were used to determine the background proliferative responses. PBMCs were immunostained with the following mAbs: anti-CD3 APC-Cy7 (BD-Pharmingen, San Diego, CA), anti-CD4 PerCP-Cy5.5 (BD-Pharmingen, San Diego, CA), anti-CD8 APC or AmCyan (BD-Pharmingen, San Diego, CA). Stained and fixed cells were acquired on an LSRI instrument using CellQuest software (BD Biosciences) and analyzed with FlowJo software (Tree Star, Ashland, OR). The level of CXCR3 was assessed using antibodies specific for CXCR3, CCR7, IFN-γ, CD3, CD8, and CD4 (BD-Pharmingen, San Diego, CA).

### Cellular activation assay

Vaccine-induced cellular activation was examined using a flow-based activation assay. Briefly, frozen PBMC samples collected before the first immunization (pre-bleed) and after the fourth (post-immunization) were thawed, rested overnight in complete medium, stained, and then analyzed by flow cytometry. The overall change in total T-cell activation on a per animal basis was determined in the absence of ex vivo stimulation by the difference in T-cell surface expression of HLA-DR between the pre-bleed and the post-immunization samples. Immunostaining of PBMCs was performed using LIVE/DEAD® Fixable Violet Dead Cell Stain Kit for 20 min at RT (Invitrogen) per the manufacturer's instructions and then washed and stained for 30 min at 4°C with PBS containing 1% FBS (1% PBS) including the following Abs: Qdot® 655-conjugated anti-CD4 (clone 19Thy-5D7; NIH), Qdot® 605-CD45RA (clone MEM-56; Invitrogen), Alexa Fluor®700-conjugated HLA-DR (clone L243; BioLegend, San Diego, CA), AmCyan-CD8 (clone SK1; BD) and PE-Cy™5-CD95 (clone DX2; BD). Next, after three washes with PBS, intracellular staining was performed using the BD Cytofix/Cytoperm™ kit and APC-Cy™7-CD3 (clone SP34-2; BD) in perm/wash buffer for 30 min at 4°C. Following staining, cells were washed and fixed with 1% paraformaldehyde. Data was collected using a LSRII flow cytometer (BD) and analyzed using FlowJo software (Tree Star, Ashland, OR).

### RNA extraction, synthesis of cRNA probes, and microarray hybridization

Total RNA for microarray analysis was isolated according to the manufacturer's protocol and further purified with an RNeasy Mini Kit (catalog no. 74104; Qiagen). Microarray analysis was performed on RNA from PBMCs taken from the animals at eight months post-vaccination and at 10 days post-SIV challenge. PBMC samples were isolated and stimulated for 24 h *in vitro* with either a SIV pol peptide pool or R10 control. This method of stimulation was used to elicit gene expression reflecting specific memory response to the SIV pol antigen. Microarray analysis was performed using Agilent RM 4×44 K microarrays using the manufacturer's one-color analysis protocol. Microarrays were performed on both SIV pol and R10 stimulated samples. To gauge the gene expression of each animal in response to SIV pol stimulation, gene expression data for each animal was calculated as the ratio of its own R10 stimulated gene expression at the concurrent time point. Thus, expression values reflect SIV pol-specific transcriptional response.

### Microarray data and functional analysis

Differential gene expression between the three groups was determined using a Textbook one-way analysis of variance (ANOVA) at the gene sequence level (ANOVA unadjusted *P*≤0.01; selected for genes with a P-value<0.05 in at least three groups; Rosetta Resolver, Rosetta Biosoftware). Pair-wise differences in expression between groups at each time point were determined by a post-hoc test of the ANOVA results (Students-Newman-Keuls adjustment, *P*≤0.1).

Functional analysis of statistically significant gene expression changes was performed using Ingenuity Pathway Analysis (IPA; Ingenuity Systems), which analyzes gene expression data in the context of known biological responses and regulatory networks. Differentially expressed gene sequences were imported into IPA software using the human GenBank accession numbers.

The biological functions and pathways that were most significantly associated with the sets of gene sequences that were differentially expressed between groups were assigned an enrichment P-value adjusted for multiple testing with a Benjamini-Hochberg test correction. For targeted assessment of specific functions, the Fisher's exact test was used to determine the probability that each biological function assigned to that data set was due to chance alone. Heatmaps for gene expression were created using Spotfire DecisionSite 9.1.1. All gene expression data described here are publicly available at http://viromics.washington.edu.

### Assessment of CXCL9 and CXCL10

A set of PBMCs obtained 8 months post the last vaccination were defrosted and stimulated *in vitro* with pol antigen for 24 h. Supernatants were harvested and tested by ELISA (R and D Systems, Minneapolis, MN) to determine the secretion of CXCL9 and CXCL10 protein.

### MHC Class I genotyping

Comprehensive sequence based MHC-I genotyping was performed using a combination of Sanger sequencing and pyrosequencing methods as previously described [43], [44]. Briefly, total cellular RNA was isolated from PBMC using the MagNA Pure LC RNA Isolation Kit (Roche Applied Sciences, Indianapolis, IN). cDNA was generated from RNA using the SuperscriptIII First-Strand Synthesis System (Invitrogen, Carlsbad, CA) and used as a template for PCR amplification with Phusion High-Fidelity Polymerase (New England BioLabs, Ipswich, MA) and MHC-I specific primers as described previously [43], [44] PCR products were gel purified using the MinElute Gel Extraction Kit (Qiagen, Valencia, CA). For Sanger sequencing, these products were cloned into bacteria using the Zero Blunt TOPO PCR Cloning Kit with TOP10 Chemically Competent E. coli (Invitrogen, Carlsbad, CA). 96–192 transformed bacterial colonies per animal were grown overnight in LB+50 ug/ml kanamycin; plasmid DNA was subsequently isolated using the Perfectprep Plasmid 96 Vac Bind Kit (5 PRIME, Gaithersburg, MD). Sanger sequencing reactions covering the highly variable peptide-binding domain encoded by MHC-I exons 2 & 3 were performed using sequence-specific PCR primers described previously [44] with the DYEnamic ET Terminator Cycle Sequencing Kit (GE Healthcare, Piscataway, NJ), and analyzed using an Applied Biosystems 3730xl Genetic Analyzer (Applied Biosystems, Foster City, CA). A subset of 5 animals was also genotyped using Roche/454-pyrosequencing of a universal diagnostic 190 bp PCR amplicon in MHC-I exon 2. This amplicon was amplified from cDNA, gel purified, quantified, normalized, and pooled as previously described23 and run on a Genome Sequencer FLX instrument at the 454 Sequencing Center (454 Life Sciences, Branford, CT) or at the University of Illinois at Urbana-Champaign High-Throughput Sequencing Center (Urbana, IL). Sequence analysis was performed using CodonCode aligner (CodonCode Corporation, Deham, MA) and Lasergene 8 (DNA Star, Madison, WI). Assembled contigs were compared to an in-house database of known rhesus macaque MHC-I alleles using BLASTN.

### Statistics

Two-tailed Mann-Whitney tests were performed using Prism Graphpad Software to compare IFN-γ ELISpots, T-cell proliferation, memory cell percent, and viral loads. P-values that were <0.05 were considered a significantly different.

## Results

### Immunization induces robust SIV-specific responses

**Figure 1:**
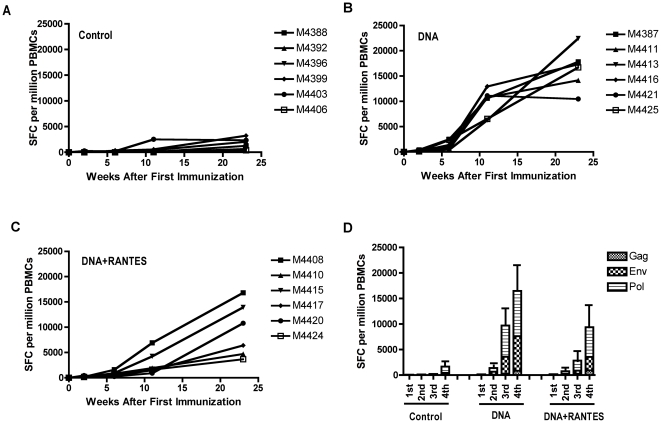
Immunization induces robust SIV-specific responses. PBMCs were isolated two weeks after each immunization and SIV-specific IFN-γ responses were assessed by standard ELISpot. Average levels of IFN-γ producing cells for the Control (A), DNA (B), and DNA+RANTES (C) groups after stimulation with SIV peptides (gag, env, or pol) are displayed. (D) Average responses per vaccine group for the individual Ags following each successive immunization. Error bars represent SD.

To evaluate the level of vaccine-induced immunity, we assessed the IFN-γ response to SIV gag, env, and pol by standard ELISpot assay (Fig. 1). PBMCs were isolated two weeks after each immunization and the cells were stimulated with pooled SIV gag, env, and pol peptides. Ag-specific responses were augmented with each subsequent immunization in the vaccinated groups, while little or no response above background was detected in the naïve group. After the fourth and final immunization, the average number of SIV gag-specific IFN-γ producing cells was similar between the two immunized groups; 839 SFCs were detected in the DNA alone group and 922 SFCs in the DNA+RANTES group. However, for both SIV env- and pol-specific IFN-γ responses, the DNA group induced higher levels than in the DNA+RANTES group; env responses averaged 6,872 and 2,631 SFCs per million PBMCs, respectively, and pol responses were 8,867 and 5,825 SFCs, respectively. Therefore, while DNA vaccination elicited robust IFN-γ responses in both groups of animals, immune responses to the SIV env and pol Ags were greater in the DNA vaccine alone group than in the RANTES-adjuvanted group.

**Figure 2:**
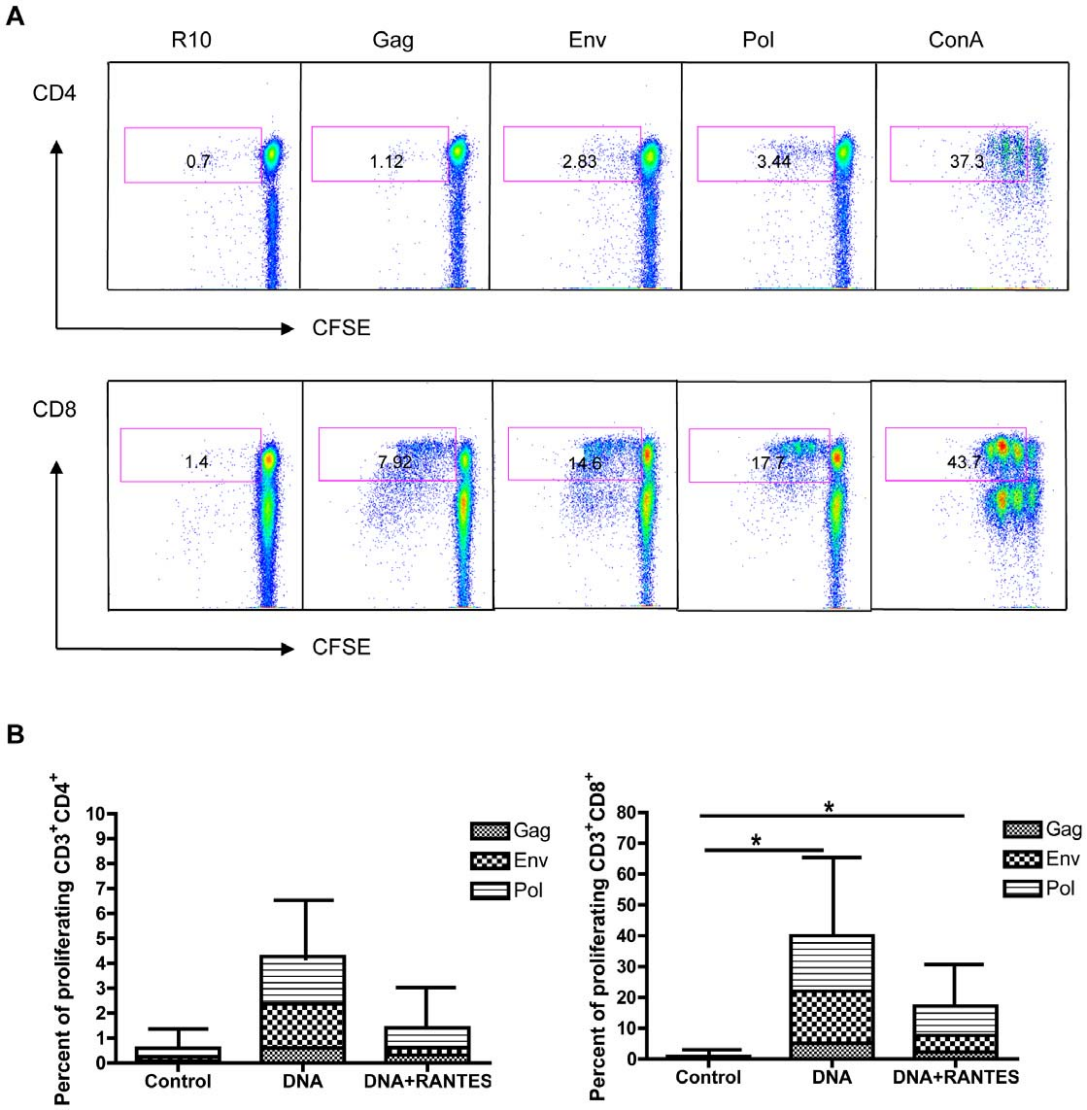
Proliferative capacity of SIV-specific T-cells in vaccinated animals. PBMCs from DNA-vaccinated RMs were isolated 4 weeks following the final immunization and analyzed for proliferative capacity. (A) Proliferation by CD4+ (top) and CD8+ (bottom) T-cells from one representative animal in the DNA-vaccinated group that were incubated for 5 days in the presence of medium alone, SIV peptides (gag, env, or pol), or ConA. Pseudo-color dot plots are gated on live CD3+ cells and T-cell gates are displayed with numbers representing total percent of CFSEdim T-cells. (B) T-cell data for each peptide (medium alone values subtracted) is enumerated per immunization group and shown for CD4+ and CD8+ T-cells. Error bars represent SD; *P<0.05 per the unpaired t test.

We next investigated the ability of vaccine-induced SIV-specific T-cells to proliferate upon antigen stimulation. PBMCs were isolated from RMs four weeks after the fourth immunization, labeled with CFSE, and then stimulated for 5 days with growth medium alone, SIV peptides, or ConA. T-cell proliferation was measured by flow cytometry, which showed that both CD4+ and CD8+ T-cell proliferative responses were higher in the DNA alone group compared to those from the DNA+RANTES group (Fig. 2). In addition, higher average proliferative responses were observed in the CD8+ compartment and that similar to ELISpot data, the env- and pol-specific responses were more extensive than the gag-specific response. These data show that DNA vaccination generates SIV-specific immunity that is capable of proliferation upon restimulation and that these responses were larger in animal receiving the DNA vaccine alone than in those that were adjuvanted. Altogether, these data demonstrate differences in the magnitudes of SIV-specific immunity generated by immunization with the DNA vaccine alone or in combination with the RANTES molecular adjuvant as measured by standard immunological assays.

**Figure 3:**
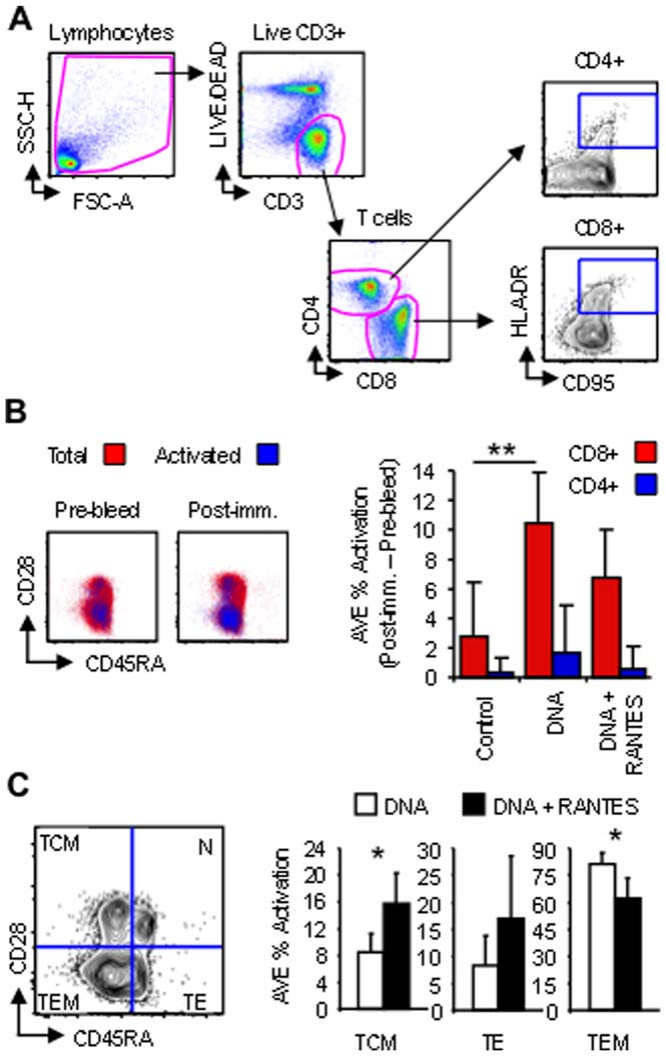
RANTES increases central memory CD8+ T formation during DNA vaccination. PBMCs isolated before the first immunization (Pre-bleed) and 2 weeks following the fourth (Post-imm.) were stained without ex vivo stimulation and analyzed by flow cytometry. (A) Strategy showing gating of total lymphocytes, live CD3+ cells, CD4+ and CD8+ T-cells, and activated cells (HLA-DR+CD95+). (B) Vaccine-induced activation from before (Pre-bleed) and after immunization (Post-imm.) are shown as dot plots with total CD8+ cells (red) overlayed by activated ones (blue) as a function of CD28 and CD45RA. The average percentage of activation per group of animals (Post-imm. – Pre-bleed) is displayed to the right for total CD8+ (red bars) and CD4+ (blue bars) T-cells. (C) Activated memory T-cell subsets were determined by gating as a function of CD28 and CD45RA, which differentiate naïve (N; CD28+CD45RA+), effectors/terminally differentiated effectors (TE; CD28−CD45RA+), central memory (TEM; CD28+CD45RA−) and effector memory (TEM; CD28−CD45RA−). The average percentage of activated cells for each phenotype is enumerated on right for DNA (white bars) versus DNA+RANTES (black bars) vaccinated animals. Graphs are pseudo-color and contour plots with outliers shown and data are from a representative animal. Error bars represent SD; *P<0.05, and **P<0.005 per the unpaired t test.

Since standard immunological assays evaluating Ag-specific immune responses using *ex vivo* stimulation may be limiting, we expanded our analysis of vaccine-induced immunity by examining the total level of cellular activation following immunization in the absence of *ex vivo* stimulation and primary cell culture. PBMC samples from before (Pre-bleed) and after the fourth immunization (Post-imm.) were stained and analyzed by flow cytometry to determine the overall change in total T-cell activation after vaccination (Fig. 3). Since HLA-DR, major histocompatibility complex class II cell surface receptor, molecules are up-regulated on the surface of T-cells in response to signaling, we gated HLA-DR+CD95+ T-cells to assess changes in T-cell activation after vaccination (Fig. 3A). Increases in vaccine-induced activation for CD4+ and CD8+ T-cells was determined for each animal by subtracting basal activation levels during the Pre-bleed from levels after the fourth immunization (Fig. 3B). Little increase in T-cell activation was observed on average for the control group; average activation levels for the control group were likely increased due to animal M4399, which exhibited an increase in CD8+ T-cell activation of almost 10% (data not shown) and may likely be the result of a common infection. However, vaccination induced measurable increases in the overall levels of CD4+ and CD8+ T-cell activation in both the DNA and the DNA+RANTES groups; vaccination with DNA alone induced a significant (*P*<0.005) 3.7-fold increase in the average percentage of CD8+ T-cell activation to a value of 10.4% while the DNA+RANTES vaccine led to a 2.4-fold increase in activation to a value of 6.8%. However, no significant increase in the level of CD4+ T-cell activation was observed in either vaccinated group. When the phenotype of activated T-cells was investigated by staining with CD28 and CD45RA, which differentiates naïve (CD28+CD45RA+), effectors/terminally differentiated effectors (CD28−CD45RA+), central memory (CD28+CD45RA−) and effector memory (CD28−CD45RA−), the DNA+RANTES vaccinated animals were observed to have greater percentages of activated central memory CD8+ T-cells on average (1.9-fold higher), but lower percentages of effector memory cells (1.3-fold lower) when compared with DNA vaccinated RMs (Fig. 3C). While no significant difference in CD4+ central memory or effectors between the vaccinated groups was found, there was a 2.2-fold higher percentage of effector memory cells in the DNA-vaccinated animals on average (data not shown). Altogether, these data show that vaccination induced measurable amounts of activation in the T-cell compartment when estimated longitudinally in the absence of *ex vivo* stimulation, but that inclusion of the RANTES adjuvant during vaccination shifted the activation phenotype of memory cells from effector to the central memory subset. Therefore, while DNA vaccination alone induced a greater amount of SIV-specific effectors as measured by standard immunological assays, DNA vaccination adjuvanted by RANTES generated a greater percentage of central memory CD8+ T-cells capable of an activated phenotype following the final immunization.

### DNA immunization with RANTES correlates with control of SIV replication following challenge

**Figure 4:**
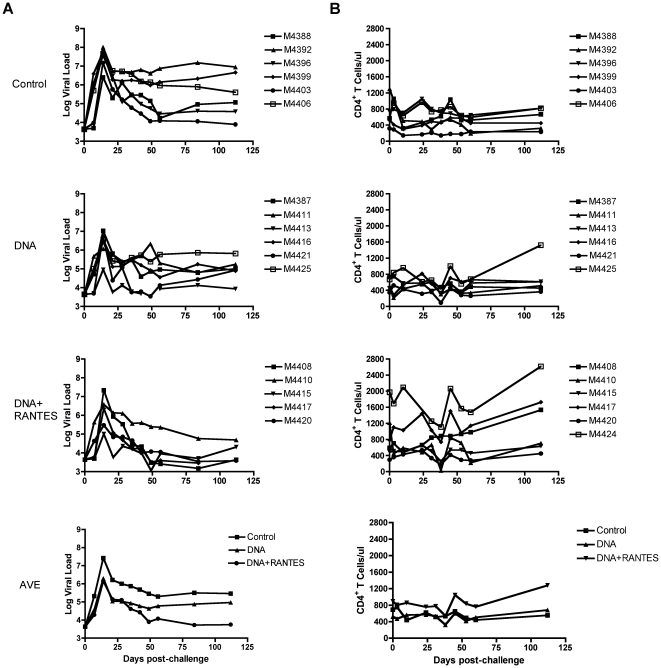
Viral load and CD4 counts in RMs after viral challenge. Viral load was measured at various time points in RMs challenged with 25 MID of SIVmac251. (A) Log viral load is presented for each animal in the Control, DNA, and DNA + RANTES groups. The average of viral loads for each group is also displayed (bottom left). (B) Number of CD4+ T-cells in macaques following challenge as measured by flow cytometry and the average number of CD4 T-cells/µl blood for each group is shown (bottom right).

Animals were challenged eight months following the fourth immunization with 25 monkey infectious doses (MID) of SIVmac251 by the intrarectal route. The average viral load in the control group at 2 weeks after challenge (peak viral load) was 7.4 logs (Fig. 4A). The average peak viral load of the DNA group and the DNA+RANTES group was 6.3 logs and 6.1 logs, respectively, and both were significantly lower than the viral load the control group (*P* = 0.007 and *P* = 0.01). However, the viral loads in DNA+RANTES group remained significantly lower than the control group at all time points measured following the peak of viremia (*P*<0.05). In addition, the animals that received DNA+RANTES exhibited a significantly lower viral load at day 84 and 112 as compared with the DNA group (*P*<0.05).

Previous SIV challenge studies have identified several MHC-I alleles that are associated with natural control of viral replication [45]–[49]. To evaluate the potential contribution of host MHC-I genetics in this study, we performed comprehensive sequence-based typing of all of the animals retrospectively (Table 2). Post-hoc analysis revealed that there were four animals that expressed the protective Mamu-B*003 allele, two in the DNA+RANTES group and one in the DNA group. An animal in the DNA+RANTES group was found to have a class I allele of the Mamu-B*017 lineage. Although we did observe significantly lower viral loads in the animals with protective haplotypes at the end of the study (p = 0.033), there was no statistical difference in peak (p = 0.968) and set point (p = 0.161) viral loads. Thus, these protective alleles may have contributed to control of viral replication during chronic infection, but they do not appear to correlate with a vaccine effect on control of viral replication observed early after infection.

**Table 2:** MHC haplotype for rhesus macaques.

Group	Sanger or 454 Typing	MHC Haplotype Associated with SIV Control	Animal ID	Number of Sequences Evaluated	Number of Unique MHC-I Alleles	B*003 Lineage	B*017 Lineage
Control	S	N	M4388	71	8		
Control	S	N	M4392	107	10		
Control	S	N	M4396	65	9		
Control	S	N	M4399	73	10		
Control	S	N	M4403	92	12		
Control	S	N	M4406	148	9		
DNA	S	N	M4387	44	7		
DNA	S	N	M4411	117	12		
DNA	S	Y	M4413	38	4	34.2	
DNA	454	N	M4416	1083	17		
DNA	S	N	M4421	110	6		
DNA	S	N	M4425	147	6		
DNA+RANTES	S	Y	M4408	100	12	31.0	
DNA+RANTES	S	N	M4410	126	11		
DNA+RANTES	S	N	M4415	96	11		
DNA+RANTES	S	N	M4417	68	6		
DNA+RANTES	S	Y	M4420	141	9	28.4	
DNA+RANTES	454	Y	M4424	780	21		12.1

Summary of comprehensive MHC class I genotypes of Chinese-origin rhesus macaques as determined by Sanger-based and Roche/454 pyrosequence-based typing. MHC class I sequences from each animal were compared to an in-house database of all known Mamu sequences using BLASTN. Total numbers of sequences evaluated and unique MHC-I alleles detected for each animal are listed. Pyrosequencing provided significantly greater depth of coverage, but the method used was still being established at the time the genotyping was performed and as such, most animals were typed using Sanger-based methods. Animals observed with MHC-I alleles or haplotypes associated with control of SIV replication (Mamu-B*003 and Mamu-B*017) are indicated by shading. The percentage of sequence reads corresponding to a given protective allele lineage in each animal are shown in burgundy. Other alleles associated with control, such as the Indian-origin rhesus macaque alleles Mamu-A1*001, Mamu-B*008 or Mamu-B*047, were not observed in this cohort. Only alleles with known positive association to SIV control are displayed here; there is always the potential that positive associations to control will be identified for other alleles observed in this cohort in the future, since most alleles expressed by Chinese-origin rhesus macaques have not been studied for correlations to SIV disease outcome.

Since depletion of CD4^+^ T-cells from mucosal sites and blood is a hallmark of HIV and SIV infection [50]–[53], we tested the CD4 T-cell counts in the blood of RMs after challenge. The CD4^+^ T-cell count relative to week 0 after challenge in the control group was lower than that in the DNA group and DNA+RANTES group (Fig. 4B). There was not a significant difference between them. Altogether, these data show that despite lower numbers of SIV-specific effectors following vaccination as measured by standard immunological assays, the DNA+RANTES immunized animals exhibiting greater levels of SIV-specific central memory cells controlled virus better than the DNA alone group and had higher CD4 T-cell counts.

### Vaccination is associated with differential transcriptional response to Pol stimulation in PBMCs

**Figure 5:**
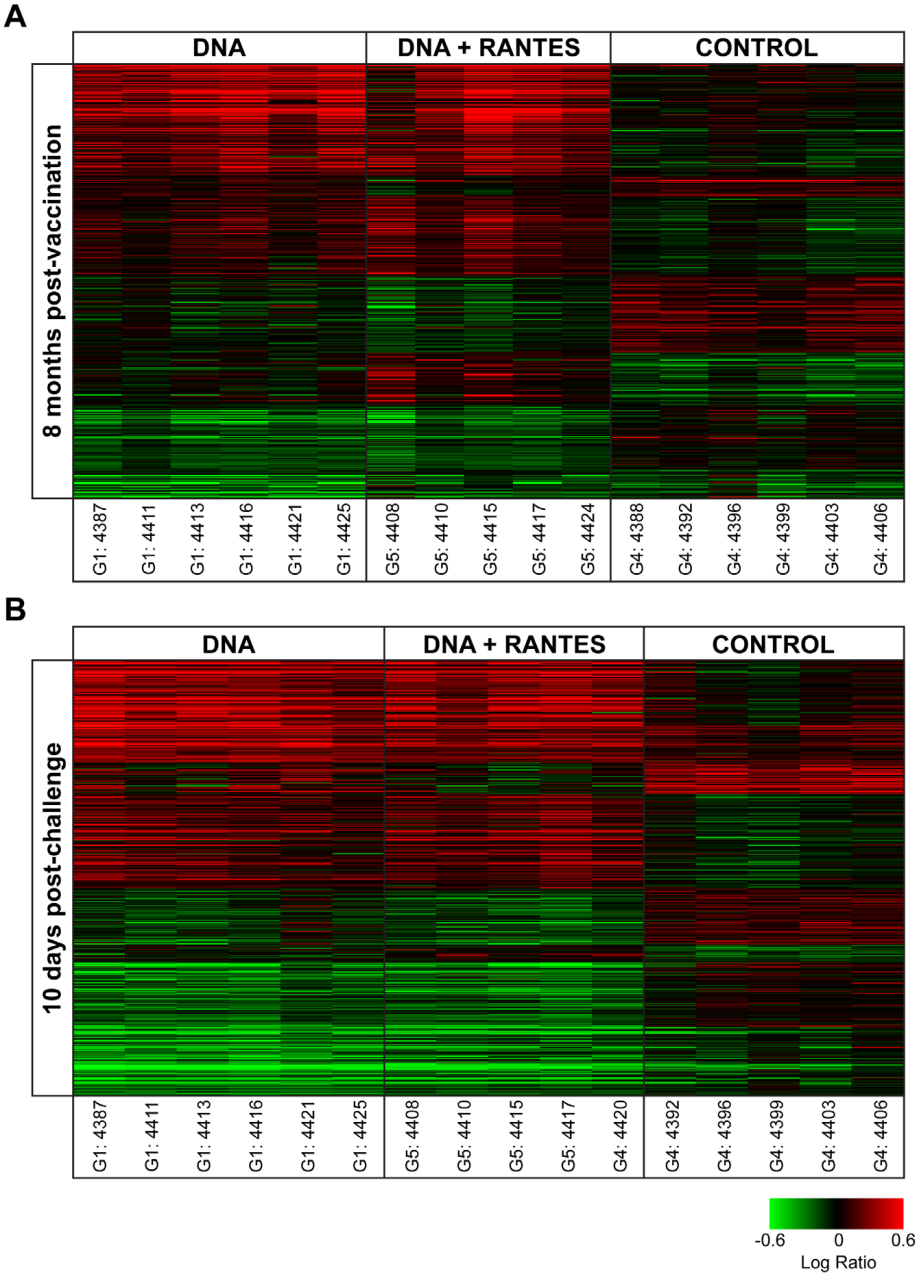
Vaccination corresponded to differential gene expression response to SIV Pol stimulation. (A) 563 gene sequences were differentially expressed at 8 months post-vaccination by ANOVA while (B) 699 gene sequences were differentially expressed among the three treatment groups at 10 days post-challenge by ANOVA. Genes shown in red have higher expression than their genetically matched mock, while genes shown in green have lower expression. Clustering was by Hierarchical algorithm with average link criteria and cosine similarity measure. Color saturation was set at +/−0.6 logratio to mock.

To explore the long-term effect of vaccination on response to SIV antigen stimulation and possible mechanisms underlying differential response to SIV challenge, gene expression in response to SIV pol peptide stimulation in fresh PBMCs was measured using DNA microarray at eight months following the last vaccination (prior to SIV challenge) and at peak viremia (10 days following SIV challenge). We applied statistical methods to distinguish treatment groups (n = 5–6 animals per group) by gene expression. At eight months following vaccination, there were 563 gene sequences that distinguished the three groups and at peak viremia there were 699 gene sequences that distinguished the three groups (Figure 5). The majority of gene expression differences were between the control group and any of the vaccinated groups, with quantitatively fewer differences distinguishing the vaccine groups. These general gene expression signatures mirror the standard immunological assays, suggesting not only the long-term impact on antigen specific response to vaccination but also relative subtly in the differences between the vaccinated groups.

### Vaccination results in an enhancement of TH1 response to stimulation at 8 months post-vaccination that is maintained following SIV challenge

**Figure 6:**
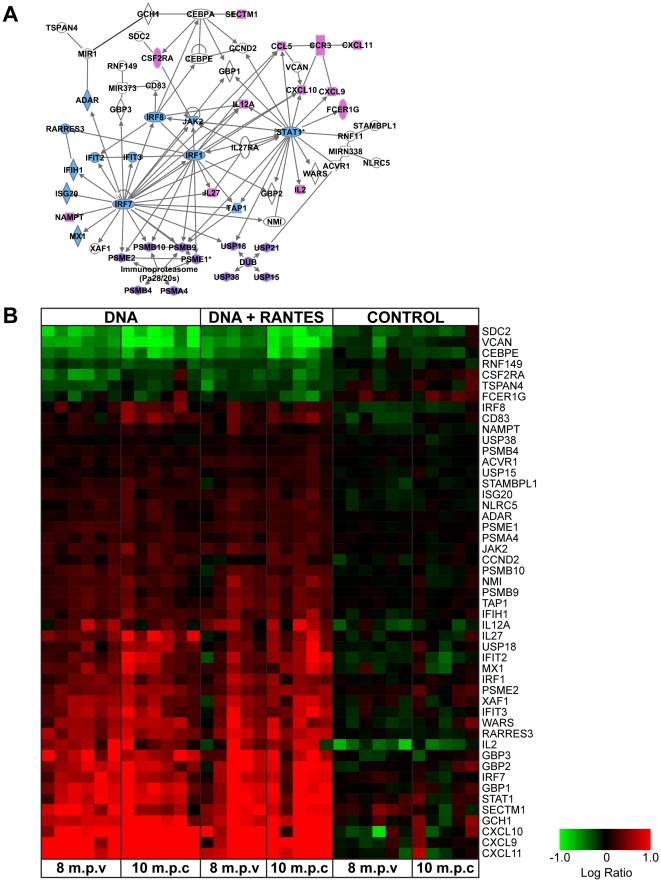
Increased expression of genes regulating IFN, Ag presentation, and chemotactic response following vaccination. (A) IPA network indicating the relationship between these differentially expressed genes. Chemokine- and cytokine-related genes (pink), IFN and anti-viral genes (blue), and genes related to protein-ubiquination and Ag presentation (purple) are shown. (B) Heatmap representing the Pol-antigen stimulated gene expression for each animal at 8 months post-vaccination (m.p.v.) and 10 days post-challenge (d.p.c.). Genes in red have higher expression than their genetically matched mock and genes in green have lower expression. Clustering was by Hierarchial algorithm with average link criteria and cosine similarity measure. Color saturation was set at +/−1.0 logratio to mock.

From these two ANOVA sets, 124 gene sequences distinguished the treatment groups at both time points. The biological functions of the genes that distinguish the treatment groups at days 0 and 10 were explored using the Ingenuity Analysis Suite (IPA). Among the genes that distinguish the groups, there was a significant enrichment of genes with functions related to both cellular and innate immune response (Table S1). Further analysis suggested that vaccination was associated with an enhanced Th1 response to pol stimulation at both pre- and post-challenge which included the increase in the expression of genes with roles in Interferon response, antigen presentation and chemokines (CXCL9, 10, 11) that activate and mobilize T-cells (Figure 6). This enhancement of interferon-related transcriptional response correlated to the increase in the number of IFN- γ producing cells as determined by the ELISpot response observed in vaccinated animals at pre- and post-challenge.

### Vaccination induces Ag-specific cells capable of secreting chemokines CXCL9 and CXCL10

**Figure 7:**
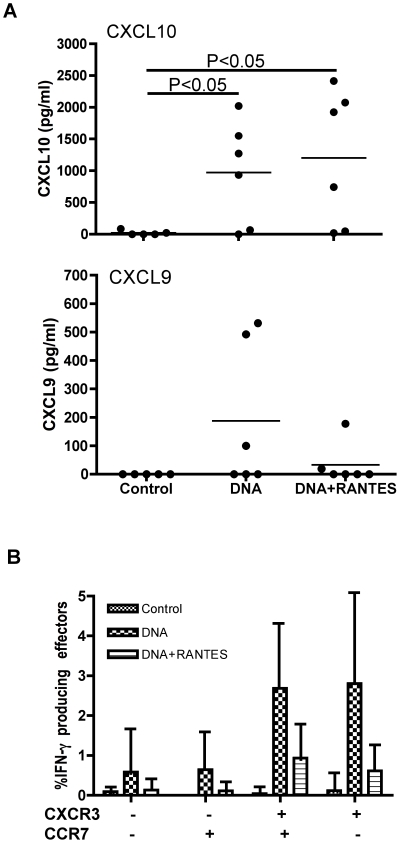
CXCL9 and CXCL10 proteins were measured by ELISA. PBMC isolated at 8 months after last immunization were defrosted and stimulated *in vitro* with SIVpol peptide for 24 h. (**A**) Supernatants were harvested and measured by ELISA for CXCL9 and CXCL10 proteins. (**B**) The level of CXCR3 was assessed using antibodies specific for CXCR3, CCR7, CD4, CD8, and CD4. PBMCs were from same time point.

To examine if differences in chemotactic gene expression correlated with an increase in protein expression, a separate set of PBMCs from 8 months after the last immunization were defrosted and stimulated *in vitro* with SIVpol antigen for 24 h. Supernatants were harvested and CXCL9 and CXCL10 proteins were measured by ELISA. We observed an average of 1,000 pg/ml and 1,100 pg/ml of CXCL10 for the DNA and the DNA+RANTES groups, respectively (Figure 7). The expression of CXCL9 protein was notably lower and less consistently detected in the PBMC of the vaccinated animals. Three of the 6 animals had responses above background in the DNA group whereas 1 of 6 animals had levels detectable above background in the DNA+RANTES group. In addition, we tested CXCR3, the receptor for CXCL9 and CXCL10 and found that the cells secreting IFN- γ were predominantly CXCR3 positive. However, when we stained for granzymeB and Tbet these CXCR3+ cells were not positive (data not shown).

### Vaccination results in an augmented cytolytic transcriptional response at 8 months post-vaccination but not at peak viremia

**Figure 8:**
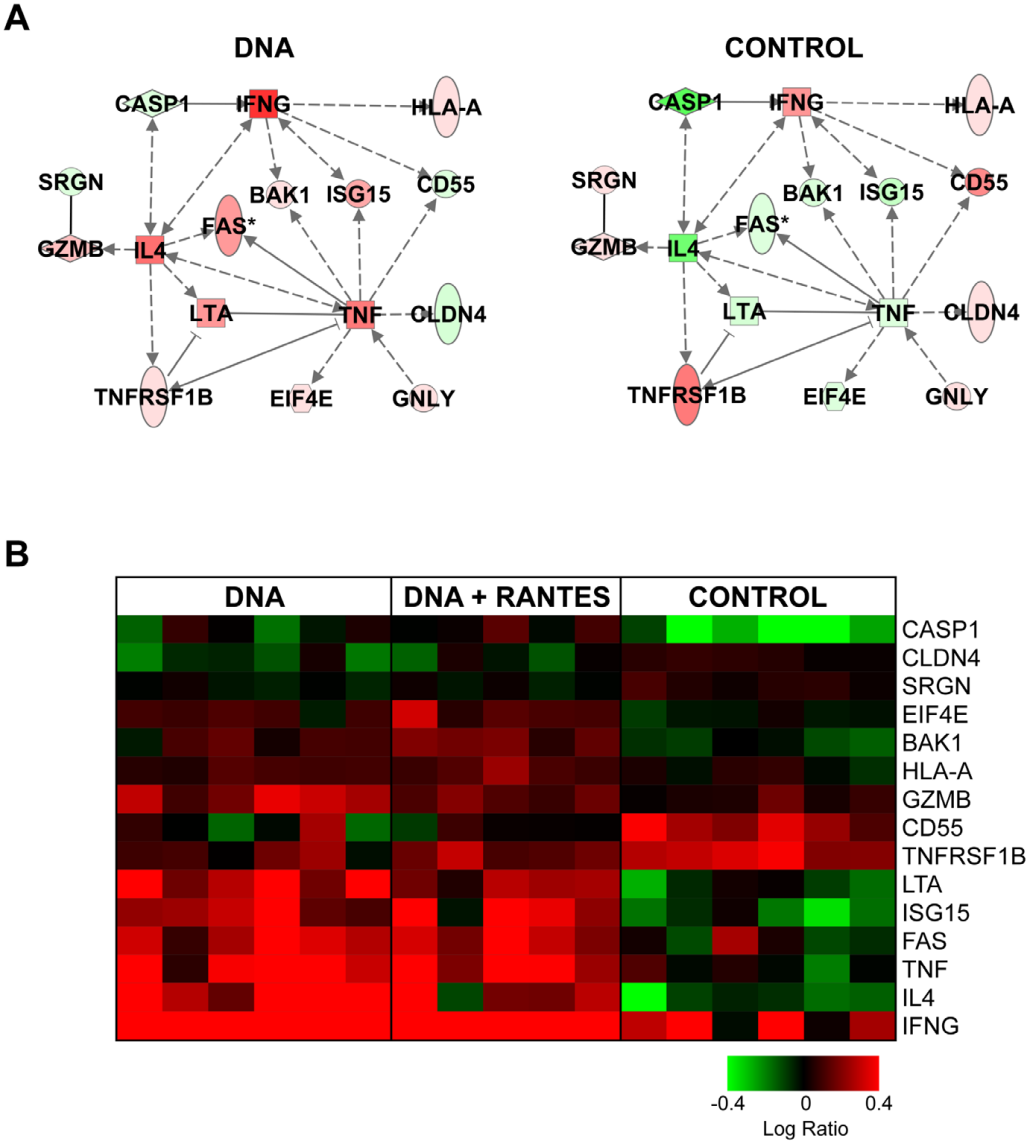
Vaccination is associated with differential regulation of cell lysis pathways at 8 months post-vaccination. (**A**) IPA networks indicate the relationship between differentially expressed genes related to cytolysis from analysis of the genes that significantly distinguished the treatment groups only at peak viremia by ANOVA. Molecule shading reflects the average logratio of expression for each group. Red represents an average increase in expression, green represents a decrease in average expression. (**B**) Heatmap indicating the expression of cytolysis genes at 8 months post-vaccination. Genes shown in red have higher expression than their genetically matched mock, gene shown in green have lower expression than their genetically matched mock. Clustering was by Hierarchial algorithm with average link criteria and cosine similarity measure. Color saturation was set at +/−0.4 logratio to mock.

Next, we examined the biological functions of the genes that distinguished the treatment groups only at 8 months post-vaccination (**Table S2**). Differences in the expression of these genes suggest a long-term transcriptional reprogramming of cells within the PBMC following vaccination that were not maintained at peak viremia. We found that vaccination was associated with an up-regulation of cytolysis-related gene expression following POL stimulation (**Figure 8**). A qualitative examination of the expression of these genes at 10 days post-challenge suggested that these genes did not pass the statistical thresholds at peak viremia because the expression of these genes was generally up-regulated in all three groups at peak viremia (**Data not shown**).

### Vaccination is associated with a decrease in cell death-related response at peak viremia

**Figure 9:**
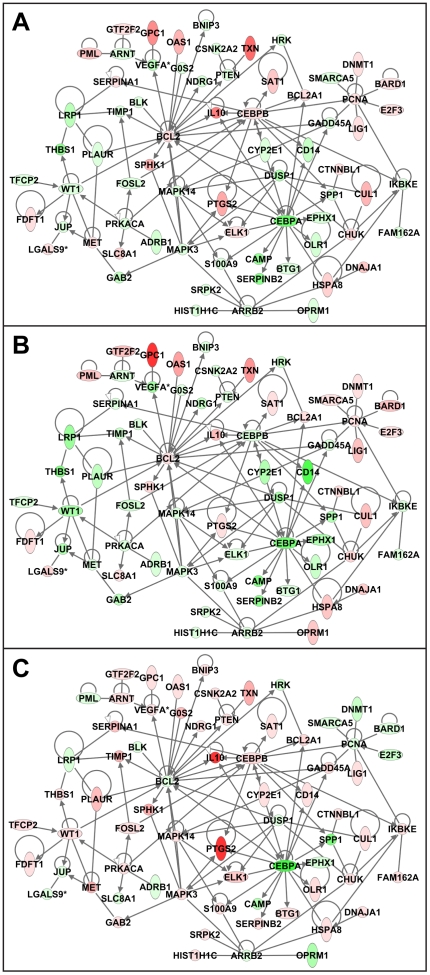
Vaccination is associated with decrease in the expression of cell death related networks at peak viremia. IPA network indicate the relationship between differentially expressed genes related to cell death as determined from analysis of the 539 genes that significantly distinguished the treatment groups only at peak viremia by ANOVA. Molecule shading reflects the average log-ratio of expression for (A) Control, (B) DNA, and (C) DNA+RANTES groups at ten days post-SIV challenge. Red represents an average increase in expression and green represents a decrease.

We next examined the genes that distinguished the groups only at peak viremia (Table S3). Differences in the expression of these genes reflect long-term reprogramming of response to SIVpol which is evident concurrent with the vast immunological insult of SIV challenge. Interestingly, at peak viremia, vaccination was associated with a decrease in the expression of cell death-related genes following SIVpol-stimulation (Figure 9).

### Adminstration of RANTES adjuvant with vaccination results in specific reprogramming of transcriptional response to SIVpol

**Figure 10:**
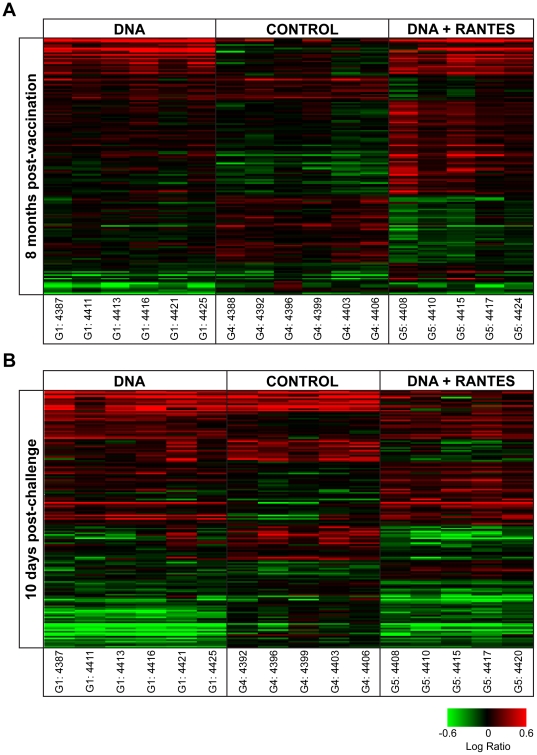
Expression for genes with unique expression in the DNA+RANTES group. (A) Expression of 156 gene sequences distinguished the DNA+RANTES group from both the DNA only and Control group at 8 months post-vaccination. (B) Expression of 159 gene sequences distinguished the DNA+RANTES group from both the DNA only and Control group at 10 days post-challenge. Genes shown in red have higher expression than their genetically matched mock, gene shown in green have lower expression than their genetically matched mock. Clustering was by Hierarchial algorithm with average link criteria and cosine similarity measure. Color saturation was set at +/−0.6 logratio to mock.

The DNA+RANTES group exhibited the greatest suppression of SIV viral load during the chronic phase of infection in this study. We next examined the genes that were uniquely expressed in the DNA+RANTES group to see if differences in transcriptional response could be correlated to the differential response to SIV challenge. Using a Student's post-hoc comparison (*P*<0.1) on the gene expression ANOVA results, we found that 156 gene sequences distinguished the DNA+RANTES group from both the DNA and Control group at 8 months post-vaccination and 159 gene sequences distinguished the RANTES group from the other two groups at peak viremia. Although there was minimal overlap between the two gene sets, the top functions related to RANTES-specific expression were very similar at both pre- and post-SIV challenge. Genes with RANTES-specific expression held functions related cell death, growth, inflammation and movement (Table S4, Figure 10). It is interesting to note that these changes in gene expression in the DNA+RANTES group not only reflect a sustained reprogramming of response to SIVpol that is unique, but these would not have necessarily been predicted by the concurrent immunological assays. Although differences in expression, such as differential expression of proliferation genes, were not born out in the immunological assays at the time, these altered responses may have as yet undetermined impact on immunological response that allows these animals to sustain their CD4 counts and suppress SIV to a greater extent during chronic infection.

## Discussion

Improved protection is the most important goal in the development of an effective vaccine for HIV-1. All of the vaccinated animals in the current study acquired SIV following a high-dose challenge with SIVmac251. However, we observed that animals in both vaccinated groups experienced a significant long-term alteration in their immune response to SIV Ags and were able to suppress viral load at peak viremia and during chronic infection. These alterations in immunity were accompanied by an increase in cellular immunity to SIV. In particular, we observed the induction of IFN-γ producing effector cells and an increase in the expression of interferon signaling pathway genes in vaccinated animals. An increase in the stimulated expression of genes related to chemotaxis including CXCL9, CXCL10 and CXCL11 were also observed. Subsequent analysis of protein levels of CXCL9 and CXCL10 secreted from Ag-activated PBMCs corroborated the microarray analysis (Figures 6b and 7). Given this data we investigated CXCR3, the receptor for CXCL9, CXCL10 and CXCL11. We found that the cells secreting IFN- γ were predominantly CXCR3 positive (Figure 7). These significant alterations in interferon, proliferation, and chemotactic responses may be partly responsible for the vaccine-associated suppression of viremia. However, the vaccine groups did not respond equally to challenge which suggests that adjuvanting altered the immune response to challenge.

RANTES co-immunization reduced viral load following challenge to a greater extent than DNA vaccination alone. However, a direct correlation between a strong immune response to SIV peptides as measured by standard immunological assays and suppression of SIV viral load was not observed. Previous studies have demonstrated that RANTES, used as an adjuvant to DNA or other vaccines, could enhance Ag-specific T-cell mediated protective immunity against HSV [54] and SHIV [55]. In contrast, we observed that the co-injection of RANTES with the vaccine actually decreased immune responses to SIV peptides when compared to those in animals receiving the DNA vaccine alone. Therefore, it is obvious from this study that others factors including the quality and/or phenotype of vaccine-specific immunity may be more important than magnitude alone.

Of particular interest was the upreglation of IL-27. IL-27 is implicated in the balance between the induction of CD8+ T cell function and Th17 helper cells. IL-27 is a heterodimeric cytokine belonging to a family of structurally related ligands that also includes IL-12, IL-23 and IL-6. Batten et al demonstrated that IL-27alpha deficient mice were hyper-susceptible to EAE and generated more IL-17 producing T helper cells [56]. IL-27 acted directly to suppress the development of IL17 producing effector T helper cells. And, recently Gosselin et al [57] found that it is the CD4+CCR6+ sub-population of T cells that are infected by HIV-1 in humans. The authors describe these lymphocyte sub-populationas as Th17 and Th1Th17 cells. Furthermore Cecchinato [58] illustrated that the Th17 cells in the gut are depleted first following SIVmac251 challenge. Finally, macaques that are capable of controlling infection have preserved Th17 cells. It is interesting that post infection, perhaps upon viral pathogenesis we no longer see and increase in IL-27 gene expression.

Indeed, flow-based activation analysis demonstrated a significantly greater percentage of vaccine-induced central memory CD8+ T-cells capable of an activated phenotype (Fig. 3) which may account for increased virus suppression upon challenge in these animals. Surface staining of lymphocyte activation without *ex vivo* stimulation showed that vaccination induced measurable amounts of activation in the T cell compartment in both vaccine groups. However, the inclusion of the RANTES adjuvant shifted the activation phenotype of memory cells from effector to the central memory subset. This indicated that, while DNA vaccination alone induced a greater amount of SIV-specific effectors, DNA vaccination adjuvanted by RANTES generated a greater percentage of central memory CD8+ T cells capable of an activated phenotype following the final immunization. According to the model proposed by Lanzavecchia A et al, [59], protective memory is mediated by effector memory T cells (T_EM_) that migrate to inflamed peripheral tissues and display immediate effector function, whereas reactive memory is mediated by central memory T cells (T_CM_) that home to T cell areas of secondary lymphoid organs, have little or no effector function, but readily proliferate and differentiate to effector cells in response to antigenic stimulation. This again suggests that the antigen-specific lymphocytes induced by the co-injection of RANTES were more likely to migrate to the lymph nodes as site of viral replication. Therefore, it is clear that memory T-cell quality was more important than quantity in providing protection.

In further support of this premise, we observed that genes related to immune cell trafficking, DNA repair, and regulation of transcription had expression patterns in the DNA+RANTES group which distinguished them from the control and DNA only groups. Of particular interest was the trafficking pathway; CD69, an early leukocyte activation molecule, was up-regulated to a greater extent in the RANTES-adjuvanted group than in the DNA-vaccinated group. This molecule is an early inducible cell surface glycoprotein acquired during lymphoid activation that is involved in lymphocyte proliferation and functions as a signal-transmitting receptor in lymphocytes and natural killer cells. Shiow et al [60] demonstrated that CD69 functions downstream of IFN-alpha and -beta and possibly other activating stimuli to promote lymphocyte retention in lymphoid organs. The function of the lymphoid organs is to filter-out invading pathogens and present them to immune cells, the high levels of viremia seen following primary HIV-SIV infection most probably lead to an efficient infection of the lymphoid tissue. Once virus is trapped within the germinal centers of the lymphoid organs, cellular recruitment and activation occur as part of the normal immune response. It is possible that the lower immune response we are seeing in the periphery is a result of the RANTES-induced antigen presenting cells trafficking to the lymphoid organ more efficiently than in the DNA alone vaccine. This is also supported by data showing that the group of macaques that were co-injected with RANTES had lower numbers of these Ag-specific PBMC cells in the periphery. It is possible that the lower levels of immune cells observed in the periphery is a result of a relocation of the cells responsible for viral suppression to the sites of infection.

The robust and sustained response to SIV antigens in the DNA and DNA+RANTES groups reflects the long-term efficacy of the DNA vaccine regimen presented here. This shows that the use of highly optimized expression vectors concurrently with EP delivery can induce a robust immune response that is qualitatively different from its DNA alone counterpart. Specifically, the co-delivery of RANTES can change the profile of the immune response so that SIV load is decreased in the peripheral blood. Although direct evidence of RANTES-induced protection against HIV or SIV has yet to be demonstrated, Hadida *et al.* have previously shown that it enhances the HIV-specific cytotoxicity of CD8+ T-cells [61]. Furthermore, they suggested that RANTES-induced FasL expression could be a defense mechanism leading to the elimination of HIV-infected cells. Therefore, FasL is likely to function in concert with the HIV-suppressive activity of RANTES to block CCR5 and induce apoptosis. However, the precise relationship between RANTES and FasL for HIV-suppression is not fully understood as enhanced expression of FasL in CTLs may also contribute to pathogenesis by accelerating the decline of Fas-expressing CD4+ or CD8+ cells [62],While our data suggest that this might be due in part to differences in immune cell localization following challenge, further studies are needed to assess whether DNA vaccination combined with RANTES adjuvanting leads to a shift in immune cell populations and viral load at sites of active infection. Overall, this study illustrates the utility of nonstandard immunological assays including flow-based activation analysis and high-throughout gene expression analysis for uncovering possible novel mechanisms of protection that would have been otherwise overlooked by standard assays.

## Supporting Information

Table S1 **Top biological functions associated with genes that were differentially regulated between groups at pre- and post-SIV challenge.**
(DOC)

Table S2 **Top biological functions associated with genes that were differentially regulated between groups only at pre-SIV challenge.**
(DOC)

Table S3 **Top biological functions associated with genes that were differentially regulated between groups only at peak viremia following SIV challenge.**
(DOC)

Table S4 **Top biological functions associated with genes that had unique expression in the DNA+RANTES group.**
(DOC)
