# Supplementary material for: Long-Term Programming of Antigen-Specific Immunity from Gene Expression Signatures in the PBMC of Rhesus Macaques Immunized with an SIV DNA Vaccine
Source: PLoS One. 2011 Jun 20;6(6):e19681. doi: 10.1371/journal.pone.0019681 (PMC3119060; doi:10.1371/journal.pone.0019681)
Supplement: Table S1 — Top biological functions associated with genes that were differentially regulated between groups at pre- and post-SIV challenge. (DOC) [file pone.0019681.s001.doc]

| **Table S1.** Top biological functions associated with genes that were differentially regulated between groups at pre- and post-SIV challenge. | | | |
| --- | --- | --- | --- |
| **Function Annotation** | **B-H p-value** | **Molecules** | **# Molecules** |
| immune response | 0.000007 | APOL1, C4B, CD83, CEBPE, CXCL9, CXCL10, CXCL11, FCER1G, GBP2, HAVCR2, IFIH1, IL2, IL27, IL12A, IRF7, IRF8, MX1, PSMB9, PSME1, PSME2, SECTM1, SLC11A1, STAT1, TAP1 | 24 |
| developmental process of leukocytes | 0.000067 | ADAR, CD83, CEBPE, CSF2RA, CXCL10, CXCL11, FCER1G, HAVCR2, IFIH1, IL2, IL27, IL12A, IRF1, IRF7, IRF8, JAK2, LGALS1, PSMB9, RARRES3, STAT1, XCL1 | 21 |
| response of leukocytes | 0.000067 | CXCL9, CXCL11, DAPP1, HAVCR2, IL2, IL27, IL12A, PSMB10, RARRES3 | 9 |
| developmental process of blood cells | 0.000067 | ADAR, CCND2, CD83, CEBPE, CSF2RA, CXCL10, CXCL11, FCER1G, HAVCR2, IFIH1, IL2, IL27, IL12A, IRF1, IRF7, IRF8, JAK2, LGALS1, PSMB9, RARRES3, STAT1, XCL1 | 22 |
| response of lymphocytes | 0.000075 | CXCL9, CXCL11, DAPP1, HAVCR2, IL2, IL27, IL12A, PSMB10 | 8 |
| replication of virus | 0.000091 | ADAR, BST2, CXCL10, IFIH1, IL2, IL12A, ISG20, MX1, RARRES3, STAT1 | 10 |
| activation of interferon-stimulated response element | 0.000112 | IRF1, IRF7, IRF8, RARRES3, TBKBP1 | 5 |
| developmental process of Th1 cells | 0.000137 | HAVCR2, IL2, IL27, IL12A, IRF1, IRF8, STAT1 | 7 |
| T cell response | 0.000216 | CXCL9, CXCL11, HAVCR2, IL2, IL27, IL12A, PSMB10 | 7 |
| maturation of blood cells | 0.000216 | CCND2, CD83, CEBPE, IFIH1, IL2, IRF1, IRF8, JAK2, RARRES3, STAT1 | 10 |
| chemotaxis of granulocytes | 0.000334 | CSF2RA, CXCL9, CXCL10, FCER1G, GRK6, IL2, LGALS1, XCL1 | 8 |
| antiviral response | 0.000343 | IFIH1, IRF1, IRF7, ISG20, MX1, RARRES3, STAT1 | 7 |
| maturation of antigen presenting cells | 0.000366 | CD83, CEBPE, IFIH1, IRF1, IRF8, RARRES3, STAT1 | 7 |
| inflammatory response | 0.000431 | APOL3, CEBPE, CSF2RA, CXCL9, CXCL10, CXCL11, FCER1G, GRK6, IL2, JAK2, LGALS1, NMI, SLC11A1, STAT1, XCL1 | 15 |
| differentiation of Th1 cells | 0.000431 | IL2, IL27, IL12A, IRF1, IRF8, STAT1 | 6 |
| growth of cells | 0.000431 | ACTN1, ADAR, CCND2, CDA, CEBPE, CSF2RA, CXCL10, DCN, FCER1G, GBP2, HAS1, HAVCR2, HNRNPAB, IFIT3, IL2, IL27, IL12A, IRF1, IRF8, JAK2, LGALS1, MX1, PSMB10, PSME2, RASL10A, SDC2, STAT1, TAP1, VCAN | 29 |
| response of cells | 0.000431 | CSF2RA, CXCL9, CXCL11, DAPP1, HAVCR2, IFIH1, IL2, IL27, IL12A, JAK2, PSMB10, RARRES3, STAT1 | 13 |
| developmental process of antigen presenting cells | 0.000614 | CD83, CEBPE, CSF2RA, IFIH1, IRF1, IRF7, IRF8, RARRES3, STAT1 | 9 |
| proliferation of blood cells | 0.000640 | CCND2, CD83, CSF2RA, DCN, HAVCR2, IL2, IL27, IL12A, IRF1, IRF8, ISG20, JAK2, LGALS1, STAT1, TP53INP1, XCL1 | 16 |
| cell movement of granulocytes | 0.000640 | C4B, CSF2RA, CXCL9, CXCL10, FCER1G, GRK6, IL2, IL27, LGALS1, XCL1 | 10 |
| antiviral response of organism | 0.000714 | IFIH1, IRF7, ISG20, MX1, RARRES3, STAT1 | 6 |
| fibrosis | 0.000714 | CXCL9, CXCL10, CXCL11, IFIT3, IL2, IRF7, JAK2, MX1, PSMB9, STAT1 | 10 |
| incorporation of guanosine 5'-O-(3-thiotriphosphate) | 0.000742 | CXCL9, CXCL10, CXCL11 | 3 |
| apoptosis of blood cells | 0.000765 | CSF2RA, DCN, FCER1G, IL2, IL12A, IRF1, IRF8, JAK2, LGALS1, NAMPT, STAT1, XCL1 | 12 |
| infectious disorder | 0.000866 | ACTN1, CD83, CEBPE, CSF2RA, CXCL9, CXCL10, CXCL11, DUSP16, FCER1G, HNRNPAB, IFIH1, IL2, IL27, IL12A, IRF1, IRF8, KPNB1, PAK3, PSMB4, PSME2, RARRES3, SLC11A1, SP110, ST3GAL5, STAT1, XCL1 | 26 |
| Biological functions were determined by Ingenuity Pathway Analysis of the 124 gene sequences that were differentially regulated at both 8 months post-vaccination and 10 days post-SIV challenge. Benjammini-Hochberg test correction was applied to P-value for biological function annotation. | | | |
