# Supplementary material for: Long-Term Programming of Antigen-Specific Immunity from Gene Expression Signatures in the PBMC of Rhesus Macaques Immunized with an SIV DNA Vaccine
Source: PLoS One. 2011 Jun 20;6(6):e19681. doi: 10.1371/journal.pone.0019681 (PMC3119060; doi:10.1371/journal.pone.0019681)
Supplement: Table S2 — Top biological functions associated with genes that were differentially regulated between groups only at pre-SIV challenge. (DOC) [file pone.0019681.s002.doc]

| **Table S2.** Top biological functions associated with genes that were differentially regulated between groups only at pre-SIV challenge. | | | |
| --- | --- | --- | --- |
| **Function Annotation** | **B-H p-value** | **Molecules** | **# Molecules** |
| cytolysis of eukaryotic cells | 0.0017 | BAK1, CD55, CLDN4, FAS, GNLY, GZMB, IFNG, ISG15, TNF, TNFRSF1B | 10 |
| generation of dendritic cells | 0.0017 | FLT3LG, IFNG, IL4, IRF4, LTA, TNF | 6 |
| formation of leukocytes | 0.0017 | FAS, IFNG, IL4, IL7, IL12RB1, LTA, MYB, TNF, TNFRSF4 | 9 |
| cytolysis of cells | 0.0017 | BAK1, CD55, CLDN4, FAS, GNLY, GZMB, IFNG, ISG15, LTA, TNF, TNFRSF1B | 11 |
| cytolysis of cell lines | 0.0017 | CD55, CLDN4, FAS, GNLY, IFNG, ISG15, TNF | 7 |
| lysis of cell lines | 0.0026 | CD55, CLDN4, EIF4E, FAS, GNLY, IFNG, ISG15, TNF | 8 |
| lysis of cells | 0.0027 | BAK1, CASP1, CD55, CLDN4, EIF4E, FAS, GNLY, GZMB, IFNG, ISG15, LTA, TNF, TNFRSF1B | 13 |
| expansion of blood cells | 0.0027 | CCND1, CD55, FLT3LG, HOXD4, IFNG, IL4, IL7, IL9, MYB, PML, SRGN, TNF, TNFRSF4, TNFRSF1B | 14 |
| cytotoxic T lymphocyte response | 0.0027 | HLA-A, IFNG, IL4, LTA, SRGN, TNF | 6 |
| quantity of phagocytes | 0.0027 | ABCA1, CXCR6, FAS, IFNG, IL4, IL9, IL12RB1, PDE4B, PML, PTGER2, PTGES, SOAT1, TNF, TNFRSF4, TNFRSF1B | 15 |
| immune response | 0.0034 | ANXA2, APOBEC3G, BAK1, BCAP31, BTN3A3, CASP1, CD55, CD69, CD300A, CISH, CXCR4, CYBA, DOK3, EIF2AK2, ERAP1, ERAP2, FAS, FLT3LG, GNLY, GZMB, HLA-A, HLA-DQA1, HLA-DRB1, HLA-DRB3, IFNG, IL4, IL7, IL9, IL12RB1, LTA, MLF2, PTGES, RGS1, STAT2, TAPBP, TICAM1, TNF, TNFRSF4, TNFRSF14, TNFRSF1B, TREM2, TRIM22 | 42 |
| lysis of eukaryotic cells | 0.0034 | BAK1, CD55, CLDN4, EIF4E, FAS, GNLY, GZMB, IFNG, ISG15, TNF, TNFRSF1B | 11 |
| experimental hepatitis of mice | 0.0034 | FAS, IFNG, IL4, TNF | 4 |
| lysis of tumor cell lines | 0.0034 | CD55, EIF4E, FAS, GNLY, IFNG, ISG15 | 6 |
| hematological process of normal cells | 0.0043 | CXCR4, ENG, FLT3LG, IFNG, IL7, PML, TEK, TNF, TNFRSF1B | 9 |
| dermatitis | 0.0050 | CASP1, CD55, CXCR4, DUSP5, EIF2AK2, HLA-DQA1, HLA-DRB1, HLA-DRB5, IDI1, IFNG, IL4, LTA, PDE4B, TNF | 14 |
| development of myeloid cells | 0.0060 | CXCR4, FLT3LG, IL4, IL7, IL9, TNF | 6 |
| activation of antigen presenting cells | 0.0060 | ANXA2, ERAP1, FAS, HLA-DQA1, IFNG, IL4, IL7, IL9, LTA, PTGES, TICAM1, TNF, TNFRSF1B, TREM2 | 14 |
| quantity of antigen presenting cells | 0.0060 | ABCA1, CXCR6, FAS, FLT3LG, IFNG, IL12RB1, IRF4, LTA, PTGER2, PTGES, SOAT1, TNF, TNFRSF4 | 13 |
| cell death of leukocyte cell lines | 0.0060 | BAK1, FAS, FLT3LG, GZMB, HLA-DRB4, IFNG, IL4, IL7, IRF4, MAX, MYB, PLAC8, PMAIP1, TNF, TNFRSF1B, ZMYM2 | 16 |
| formation of T lymphocytes | 0.0060 | FAS, IL7, IL12RB1, MYB, TNFRSF4 | 5 |
| apoptosis of bone cell lines | 0.0060 | FAS, FLT3LG, GZMB, IL4, MAX, MYB, PLAC8, PTK2B, TNF | 9 |
| Rett syndrome | 0.0060 | BTN3A1, CXCR6, ERAP1, ERAP2, FDXR, HLA-DRB4, IFNG, MREG, NAPRT1, PDE4DIP, PTPN13, TOM1 | 12 |
| respiratory burst of monocytes | 0.0060 | IFNG, IL4, IL9, TNF | 4 |
| Biological functions were determined by Ingenuity Pathway Analysis of the 439 gene sequences that were differentially regulated at between groups at 8 months post-vaccination only as determined by ANOVA. Benjammini-Hochberg test correction was applied to P-value for biological function annotation. | | | |
