# Supplementary material for: Long-Term Programming of Antigen-Specific Immunity from Gene Expression Signatures in the PBMC of Rhesus Macaques Immunized with an SIV DNA Vaccine
Source: PLoS One. 2011 Jun 20;6(6):e19681. doi: 10.1371/journal.pone.0019681 (PMC3119060; doi:10.1371/journal.pone.0019681)
Supplement: Table S3 — Top biological functions associated with genes that were differentially regulated between groups only at peak viremia following SIV challenge. (DOC) [file pone.0019681.s003.doc]

| **Table S3.** Top biological functions associated with genes that were differentially regulated between groups only at peak viremia following SIV challenge. | | | |
| --- | --- | --- | --- |
| **Function Annotation** | **B-H p-value** | **Molecules** | **# Molecules** |
| tumorigenesis of organ | 0.0036 | BCL2, CD1D, CEBPB, CUL1, DNMT1, EPHX1, GADD45A, GHRH, MAPK14, NDRG1, PRKCB, PTEN, SERPINB2, SPP1, TIMP1, VEGFA | 16 |
| cell death of eukaryotic cells | 0.0036 | ABCB7, ABCG1, ADRB1, ARNT, ARRB2, BARD1, BCL2, BCL2A1, BLK, BNIP3, BTG1, BUB1, CAMP, CASP8AP2, CCT2, CCT7, CD14, CD22, CD1D, CEBPA, CEBPB, CHUK, CSNK2A2, CXCL3, CYP2E1, DDIT4, DFFA, DNMT1, DUSP1, E2F3, ECEL1, ELK1, EPHX1, FDFT1, G0S2, GAB2, GADD45A, GPC1, GTF2F2, GZMM, HIP1, HIST1H1C, HK1, HRK, HSPA8, IFNGR1, IKBKE, IL10, IL6R, JUP, KIF1A, KIR2DL3, LGALS9, LIG1, LRP1, LZTS2, MAPK3, MAPK14, MAPKAP1, MET, NCAM1, NDRG1, NGFRAP1, NOD1, NOL3, NRG1, OLR1, OPRM1, PCNA, PHLDA1, PLAUR, PLD1, PML, PRDX6, PRKACA, PRKCB, PTEN, PTGS2, RASD1, RBCK1, S100A9, S100P, SAT1, SERPINA1, SERPINA3, SERPINB2, SERPINB10, SIGLEC9, SLC8A1, SMARCA5, SPHK1, SPP1, SRPK2, TCF3, TFCP2, THBS1, TIMP1, TNIP1, TPP1, TXN, UBD, VDAC2, VEGFA, WT1 | 104 |
| cell death | 0.0036 | ABCB7, ABCG1, ADRB1, ARNT, ARRB2, BARD1, BCL2, BCL2A1, BLK, BNIP3, BTG1, BUB1, CAMP, CASP5, CASP8AP2, CCT2, CCT7, CD14, CD22, CD1D, CEBPA, CEBPB, CHUK, CSNK2A2, CTNNBL1, CUL1, CXCL3, CYP2E1, DDIT4, DDX19A, DFFA, DNAJA1, DNMT1, DUSP1, E2F3, ECEL1, ELK1, EPHX1, F13A1, FAM162A, FDFT1, FOSL2, FXR1, G0S2, GAB2, GABPB1, GADD45A, GPC1, GTF2F2, GZMM, HIP1, HIST1H1C, HK1, HRK, HSPA8, IFNGR1, IKBKE, IL10, IL6R, JUP, KIF1A, KIR2DL3, LGALS9, LIG1, LRP1, LY86, LZTS2, MAPK3, MAPK14, MAPKAP1, MET, NCAM1, NDRG1, NGFRAP1, NOD1, NOL3, NRG1, OAS1, OLR1, OPRM1, PAFAH1B3, PCNA, PHLDA1, PLAUR, PLD1, PML, PRDX6, PRKACA, PRKCB, PSMG2, PTEN, PTGS2, RASD1, RBCK1, S100A9, S100P, SAT1, SERINC3, SERPINA1, SERPINA3, SERPINB2, SERPINB10, SGK3, SIGLEC9, SLC8A1, SMARCA5, SPHK1, SPP1, SRPK2, TCF3, TFCP2, THBS1, TIMP1, TNFRSF12A, TNIP1, TPP1, TXN, UBD, VDAC2, VEGFA, WT1 | 121 |
| cancer | 0.0041 | AACS, ABR, ACTR10, ADAMDEC1, ADRB1, AGPAT6, AIM1, AKR1C3, ARAF, ARNT, ARRB2, BACH1, BARD1, BCL2, BNIP3, BRIP1, C10ORF54, C17ORF61, C1R, C1S, C6ORF211, CACNA1G, CAMP, CBLN1, CD14, CD22, CD276, CEBPA, CEBPB, CHUK, CNNM2, CUL1, CXCL14, CXCR7, CYP27B1, CYP2E1, DDIT4, DGAT2, DNAJA1, DNMT1, DSTN, DUSP1, E2F3, EPHX1, EPS8, EREG, FABP3, FAM124A, FAM57A, FDFT1, FOSL2, FZD4, GADD45A, GHRH, GPC1, GPRC5A, HIP1, HIST1H4H, HNMT, HRK, HSPA8, IFI27, IFNGR1, IKBKE, IL10, IL6R, IMPDH1, INSIG1, JUP, KDELR1, KDM1A, KIF1A, LIG1, MAB21L1, MAP4K4, MAPK3, MAPK14, MCM3, MET, MICALL1, MMP25, MR1, NCAM1, NDRG1, NRG1, NUDT5, OLR1, OPRM1, ORM1, PCNA, PLAUR, PLOD2, PML, PPAP2A, PPPDE1, PRKCB, PSAT1, PTEN, PTGS2, RIN1, RRP12, S100A9, S100P, SAT1, SDHD, SERPINA1, SERPINA3, SERPINB1, SERPING1, SGK3, SLC25A5, SLC7A5, SMARCD3, SPHK1, SPP1, SULT1A1, SULT1A2, TBXAS1, TCF3, TFPI, THBS1, TIMP1, TLR5, TMEM106C, TNFRSF12A, TTC22, TXN, VDAC2, VEGFA, WNT5A, WT1, XPO5 | 132 |
| developmental process of tumor | 0.0041 | ARRB2, BCL2, CEBPB, DNMT1, E2F3, GADD45A, GHRH, HSPA8, LRP1, MAPK14, MET, NDRG1, OLR1, PML, PTEN, PTGS2, SAT1, SPP1, TCF3, THBS1, TIMP1, TXN, VEGFA | 23 |
| neoplasia | 0.0041 | AACS, ABR, ACTR10, ADAMDEC1, ADRB1, AGPAT6, AIM1, AKR1C3, ARAF, ARNT, ARRB2, BACH1, BARD1, BCL2, BNIP3, BRIP1, C10ORF54, C17ORF61, C1R, C1S, C6ORF211, CACNA1G, CAMP, CBLN1, CD14, CD22, CD276, CEBPA, CEBPB, CHUK, CNNM2, CUL1, CXCL14, CXCR7, CYP27B1, CYP2E1, DDIT4, DGAT2, DNAJA1, DNMT1, DSTN, DUSP1, E2F3, EPHX1, EPS8, EREG, FABP3, FAM124A, FAM57A, FDFT1, FOSL2, FZD4, GADD45A, GHRH, GPC1, GPRC5A, HIP1, HIST1H4H, HMGN1, HNMT, HRK, HSPA8, IFI27, IFNGR1, IKBKE, IL10, IL6R, IMPDH1, INSIG1, JUP, KDELR1, KDM1A, KIF1A, LIG1, MAB21L1, MAP4K4, MAPK3, MAPK14, MCM3, MET, MICALL1, MMP25, MR1, NCAM1, NDRG1, NRG1, NUDT5, OLR1, OPRM1, ORM1, PCNA, PLAUR, PLOD2, PML, PPAP2A, PPPDE1, PRDX6, PRKCB, PSAT1, PTEN, PTGS2, RIN1, RRP12, S100A9, S100P, SAT1, SDHD, SERPINA1, SERPINA3, SERPINB1, SERPINB2, SERPING1, SGK3, SLC25A5, SLC7A5, SMARCD3, SPHK1, SPP1, SULT1A1, SULT1A2, TBXAS1, TCF3, TFPI, THBS1, TIMP1, TLR5, TMEM106C, TNFRSF12A, TTC22, TXN, VDAC2, VEGFA, WNT5A, WT1, XPO5 | 135 |
| tumorigenesis | 0.0041 | AACS, ABR, ACTR10, ADAMDEC1, ADRB1, AGPAT6, AIM1, AKR1C3, ANG, ARAF, ARNT, ARRB2, BACH1, BARD1, BCL2, BNIP3, BRIP1, BUB1, C10ORF54, C17ORF61, C1R, C1S, C6ORF211, CACNA1G, CAMP, CBLN1, CD14, CD22, CD276, CD1D, CEBPA, CEBPB, CHUK, CNNM2, CSNK2A2, CUL1, CXCL14, CXCR7, CYP27B1, CYP2E1, DDIT4, DGAT2, DNAJA1, DNMT1, DSTN, DUSP1, E2F3, EPHX1, EPS8, EREG, FABP3, FAM124A, FAM57A, FDFT1, FOSL2, FZD4, GADD45A, GHRH, GPC1, GPRC5A, HIP1, HIST1H4H, HMGN1, HNMT, HRK, HSPA8, IFI27, IFNGR1, IKBKE, IL10, IL6R, IMPDH1, INSIG1, JUP, KDELR1, KDM1A, KIF1A, LIG1, LRP1, MAB21L1, MAP4K4, MAPK3, MAPK14, MCM3, MET, MICALL1, MMP25, MR1, NCAM1, NDRG1, NRG1, NUDT5, OLR1, OPRM1, ORM1, PCNA, PLAUR, PLOD2, PML, PPAP2A, PPPDE1, PRDX6, PRKCB, PSAT1, PTEN, PTGS2, RIN1, RRP12, S100A9, S100P, SAT1, SDHD, SERPINA1, SERPINA3, SERPINB1, SERPINB2, SERPING1, SGK3, SLC25A5, SLC7A5, SMARCD3, SPHK1, SPP1, SULT1A1, SULT1A2, TBXAS1, TCF3, TFPI, THBS1, TIMP1, TLR5, TMEM106C, TNFRSF12A, TNS1, TTC22, TXN, VDAC2, VEGFA, WNT5A, WT1, XPO5 | 141 |
| apoptosis of normal cells | 0.0041 | ABCG1, ADRB1, ARRB2, BCL2, BCL2A1, BNIP3, BTG1, CAMP, CD14, CD22, CD1D, CEBPA, CEBPB, CHUK, CSNK2A2, DFFA, DUSP1, ELK1, GADD45A, HIP1, HIST1H1C, HRK, IFNGR1, IKBKE, IL10, IL6R, LGALS9, LRP1, MAPK14, MET, NGFRAP1, NOL3, NRG1, OLR1, OPRM1, PLAUR, PLD1, PML, PRDX6, PRKACA, PRKCB, PTEN, PTGS2, RBCK1, SERPINA1, SERPINA3, SIGLEC9, SLC8A1, SPHK1, SPP1, SRPK2, THBS1, TIMP1, TNIP1, TXN, UBD, VEGFA, WT1 | 58 |
| cell death of leukemia cell lines | 0.0042 | BCL2, BCL2A1, CAMP, CEBPA, DFFA, DUSP1, GZMM, HSPA8, KIR2DL3, LGALS9, LRP1, MAPK14, MET, PML, PRKACA, PRKCB, PTEN, S100A9, SPHK1, TCF3, THBS1, WT1 | 22 |
| apoptosis of eukaryotic cells | 0.0042 | ABCG1, ADRB1, ARRB2, BARD1, BCL2, BCL2A1, BLK, BNIP3, BTG1, CAMP, CASP8AP2, CCT2, CD14, CD22, CD1D, CEBPA, CEBPB, CHUK, CSNK2A2, DDIT4, DFFA, DNMT1, DUSP1, E2F3, ECEL1, ELK1, EPHX1, G0S2, GAB2, GADD45A, GZMM, HIP1, HIST1H1C, HK1, HRK, HSPA8, IFNGR1, IKBKE, IL10, IL6R, JUP, LGALS9, LIG1, LRP1, LZTS2, MAPK3, MAPK14, MAPKAP1, MET, NCAM1, NDRG1, NGFRAP1, NOD1, NOL3, NRG1, OLR1, OPRM1, PCNA, PHLDA1, PLAUR, PLD1, PML, PRDX6, PRKACA, PRKCB, PTEN, PTGS2, RASD1, RBCK1, S100A9, SAT1, SERPINA1, SERPINA3, SIGLEC9, SLC8A1, SPHK1, SPP1, SRPK2, TCF3, TFCP2, THBS1, TIMP1, TNIP1, TXN, UBD, VDAC2, VEGFA, WT1 | 88 |
| cell death of normal cells | 0.0042 | ABCG1, ADRB1, ARRB2, BCL2, BCL2A1, BNIP3, BTG1, BUB1, CAMP, CD14, CD22, CD1D, CEBPA, CEBPB, CHUK, CSNK2A2, CXCL3, DFFA, DUSP1, ELK1, EPHX1, GADD45A, GTF2F2, HIP1, HIST1H1C, HK1, HRK, IFNGR1, IKBKE, IL10, IL6R, KIF1A, LGALS9, LRP1, MAPK14, MET, NGFRAP1, NOL3, NRG1, OLR1, OPRM1, PLAUR, PLD1, PML, PRDX6, PRKACA, PRKCB, PTEN, PTGS2, RBCK1, SERPINA1, SERPINA3, SIGLEC9, SLC8A1, SMARCA5, SPHK1, SPP1, SRPK2, TFCP2, THBS1, TIMP1, TNIP1, TPP1, TXN, UBD, VEGFA, WT1 | 67 |
| proliferation of cell lines | 0.0042 | ABCB7, AKR1C3, ANG, BARD1, BCL2, BTG1, C5ORF13, CACNA1G, CAMP, CCL20, CEBPA, CEBPB, CSNK2A2, CXCL3, CXCR7, DTL, E2F3, EPS8, FDFT1, GAB2, GABARAP, GHRH, HK1, IKBKE, IL10, IL6R, JUP, LIG1, MAP4K4, MAPK3, MAPK14, MET, NRG1, NRP2, PCNA, PLAUR, PML, PRKACA, PRKCB, PTEN, PTGS2, PTP4A3, REM2, S100A9, S100P, SAT1, SERPINB2, SMARCA4, SPHK1, SPP1, THBS1, TLR5, TXN, VEGFA, WNT5A, WT1 | 56 |
| apoptosis of leukemia cell lines | 0.0054 | BCL2, BCL2A1, CAMP, CEBPA, DFFA, DUSP1, GZMM, HSPA8, LGALS9, LRP1, MAPK14, MET, PML, PRKACA, PRKCB, PTEN, S100A9, SPHK1, TCF3, WT1 | 20 |
| severe acute respiratory syndrome | 0.0056 | C3AR1, CAMP, G3BP2, HIST1H1C, HIST1H2AC, HIST1H2BK, PYGL, RAB13, S100A9, S100A12, S100P, SERPINA1, TIMP1, TKT | 14 |
| recruitment of phagocytes | 0.0062 | ARRB2, C3AR1, CAMP, CCL20, CD14, CXCL3, F13A1, IL10, IL6R, NOD1, PTEN, THBS1, TLR5 | 13 |
| neoplasia of organ | 0.0072 | CUL1, NDRG1, PRKCB, PTEN, SERPINB2, SPP1, TIMP1, VEGFA | 8 |
| cell death of tumor cell lines | 0.0084 | ABCB7, ARRB2, BARD1, BCL2, BCL2A1, BNIP3, CAMP, CASP8AP2, CCT2, CCT7, CEBPA, CEBPB, CSNK2A2, CYP2E1, DDIT4, DFFA, DUSP1, ELK1, FDFT1, G0S2, GADD45A, GPC1, GZMM, HIP1, HSPA8, IKBKE, IL10, JUP, KIR2DL3, LGALS9, LIG1, LRP1, MAPK3, MAPK14, MAPKAP1, MET, NCAM1, NDRG1, NOD1, NOL3, NRG1, PCNA, PLAUR, PML, PRKACA, PRKCB, PTEN, PTGS2, RASD1, S100A9, SAT1, SERPINB10, SPHK1, SPP1, TCF3, TFCP2, THBS1, TXN, VDAC2, VEGFA, WT1 | 61 |
| cell death of cell lines | 0.0085 | ABCB7, ARNT, ARRB2, BARD1, BCL2, BCL2A1, BLK, BNIP3, CAMP, CASP8AP2, CCT2, CCT7, CD22, CEBPA, CEBPB, CSNK2A2, CYP2E1, DDIT4, DFFA, DNMT1, DUSP1, E2F3, ECEL1, ELK1, EPHX1, FDFT1, G0S2, GADD45A, GPC1, GZMM, HIP1, HK1, HSPA8, IKBKE, IL10, JUP, KIR2DL3, LGALS9, LIG1, LRP1, LZTS2, MAPK3, MAPK14, MAPKAP1, MET, NCAM1, NDRG1, NOD1, NOL3, NRG1, PCNA, PLAUR, PLD1, PML, PRKACA, PRKCB, PTEN, PTGS2, RASD1, S100A9, S100P, SAT1, SERPINB2, SERPINB10, SPHK1, SPP1, TCF3, TFCP2, THBS1, TIMP1, TNIP1, TXN, VDAC2, VEGFA, WT1 | 75 |
| apoptosis | 0.0085 | ABCG1, ADRB1, ARRB2, BARD1, BCL2, BCL2A1, BLK, BNIP3, BTG1, CAMP, CASP5, CASP8AP2, CCT2, CD14, CD22, CD1D, CEBPA, CEBPB, CHUK, CSNK2A2, CTNNBL1, CUL1, DDIT4, DDX19A, DFFA, DNMT1, DUSP1, E2F3, ECEL1, ELK1, EPHX1, FAM162A, FXR1, G0S2, GAB2, GABPB1, GADD45A, GZMM, HIP1, HIST1H1C, HK1, HRK, HSPA8, IFNGR1, IKBKE, IL10, IL6R, JUP, LGALS9, LIG1, LRP1, LY86, LZTS2, MAPK3, MAPK14, MAPKAP1, MET, NCAM1, NDRG1, NGFRAP1, NOD1, NOL3, NRG1, OAS1, OLR1, OPRM1, PAFAH1B3, PCNA, PHLDA1, PLAUR, PLD1, PML, PRDX6, PRKACA, PRKCB, PSMG2, PTEN, PTGS2, RASD1, RBCK1, S100A9, SAT1, SERINC3, SERPINA1, SERPINA3, SGK3, SIGLEC9, SLC8A1, SPHK1, SPP1, SRPK2, TCF3, TFCP2, THBS1, TIMP1, TNFRSF12A, TNIP1, TXN, UBD, VDAC2, VEGFA, WT1 | 102 |
| recruitment of neutrophils | 0.0088 | ARRB2, C3AR1, CAMP, CCL20, CD14, CXCL3, IL10, IL6R, NOD1, PTEN, TLR5 | 11 |
| proliferation of tumor cell lines | 0.0088 | ABCB7, AKR1C3, ANG, BARD1, BCL2, CACNA1G, CCL20, CEBPA, CEBPB, CSNK2A2, DTL, EPS8, FDFT1, GHRH, HK1, IKBKE, IL10, LIG1, MAP4K4, MAPK3, MAPK14, MET, NRG1, PCNA, PLAUR, PML, PRKACA, PRKCB, PTEN, PTGS2, PTP4A3, SAT1, SERPINB2, SMARCA4, SPHK1, SPP1, THBS1, TLR5, VEGFA, WNT5A, WT1 | 41 |
| developmental process of leukemia cell lines | 0.0088 | AKR1C3, BCL2, CEBPA, CEBPB, GAB2, GNA15, IL10, MAPK14, NRG1, PML, PRKACA, PRKCB, PTEN, SERPINB2, TCF3, TIMP1, WT1 | 17 |
| quantity of lipid | 0.0088 | ABCG1, ADRB1, AGPAT6, ARF1, ARNT, BCL2, CEBPA, CEBPB, CYP27B1, CYP2E1, DGAT2, FABP3, FDFT1, GBP5, GHRH, IDH1, IL10, INSIG1, LRP1, MAG, OLR1, PITPNB, PLA1A, PLD1, PLIN2, PMCH, PRDX6, PRKCB, PTEN, PTGS2, SAT1, SPHK1, TIMP1, TNFRSF12A | 34 |
| proliferation of eukaryotic cells | 0.0092 | ABCB7, ABCG1, AKR1C3, ANG, ARNT, ATF4, ATPIF1, BARD1, BCL2, BCL2A1, BTG1, C3AR1, C5ORF13, CACNA1G, CAMP, CCL20, CD14, CD22, CD276, CD1D, CEBPA, CEBPB, CHUK, CSNK2A2, CXCL3, CXCR7, DOCK2, DTL, DUSP1, E2F3, EPS8, EREG, FDFT1, FOSL2, GAB2, GABARAP, GADD45A, GHRH, HK1, HMGN1, IFNGR1, IKBKE, IL10, IL6R, IMPDH1, JUP, LIG1, LRP1, LY86, MAP4K4, MAPK3, MAPK14, MET, NCAM1, NRG1, NRP2, NUMBL, PCNA, PLAUR, PML, PRKACA, PRKCB, PTEN, PTGS2, PTP4A3, REM2, S100A9, S100P, SAT1, SERPINA1, SERPINB2, SGK3, SLAMF7, SLC7A5, SMARCA4, SPHK1, SPP1, TCF3, TFPI, THBS1, TLR5, TNFRSF12A, TXN, VEGFA, WNT5A, WT1 | 86 |
| prostatic carcinoma | 0.0097 | ADRB1, AIM1, BCL2, C1R, DUSP1, FAM124A, FDFT1, GPC1, GPRC5A, HIP1, IL10, KDM1A, OPRM1, ORM1, PLAUR, PML, PRKCB, PSAT1, PTEN, PTGS2, S100A9, SAT1, SERPINB1, SERPING1, SMARCD3, VEGFA, WT1 | 27 |
| Biological functions were determined by Ingenuity Pathway Analysis of the 575 gene sequences that were differentially regulated at between groups at 10 days post-SIV challenge only as determined by ANOVA. Benjammini-Hochberg test correction was applied to P-value for biological function annotation. | | | |
