# Supplementary material for: Long-Term Programming of Antigen-Specific Immunity from Gene Expression Signatures in the PBMC of Rhesus Macaques Immunized with an SIV DNA Vaccine
Source: PLoS One. 2011 Jun 20;6(6):e19681. doi: 10.1371/journal.pone.0019681 (PMC3119060; doi:10.1371/journal.pone.0019681)
Supplement: Table S4 — Top biological functions associated with genes that had unique expression in the DNA+RANTES group. (DOC) [file pone.0019681.s004.doc]

| **Table S4.** Top biological functions associated with genes that had unique expression in the DNA+RANTES group | | | | |
| --- | --- | --- | --- | --- |
| **(A) 8 months post-vaccination** | | | | |
| **Function Annotation** | **B-H p-value** | **Molecules** | | **# Molecules** |
| tumorigenesis | 0.028 | ACVR1, AFF1, ANK3, ATOH1, BAK1, BAP1, CALCOCO2, CCND1, CRKRS, CSDE1, CSF2RA, CXCL9, DUSP5, EDNRB, EED, FDXR, FGFR1OP2, FOXM1, GSS, HNRNPH1, IFNG, IL4, IL7, IVD, LTA, MAX, MCM10, MT1H, MYB, OVGP1, PDE4B, PMAIP1, POLD1, PTGER2, SDC2, ST14, SYT12, TEK, TGFA, TIMM44, TPR, TWF1, ZFP36L2, ZMYM2 | | 44 |
| cell death | 0.047 | A4GALT, ACVR1, AGAP3, ATOH1, BAK1, BAP1, CCND1, CD53, CSF2RA, DTYMK, EDNRB, FDXR, FOXM1, GATAD2A, GNLY, IFNG, IL4, IL7, LTA, MAX, MCM10, MYB, PMAIP1, PTGER2, RASL10A, RDX, RNF34, SDC2, SOAT1, SOX10, TGFA, TMEM102, ZFP36L2, ZMYM2 | | 34 |
| growth of eukaryotic cells | 0.046 | AFF1, CCND1, CSF2RA, DUSP5, FDXR, FOXM1, IFNG, IL4, IL7, IL27, MAX, MYB, PTGER2, RASL10A, SDC2, TEK, TGFA, TPR, TRPM7, ZMYM2 | | 20 |
| movement of eukaryotic cells | 0.047 | ACVR1, ATOH1, CCND1, CSF2RA, CXCL9, EDNRB, FOXM1, GNLY, IFNG, IL4, IL7, LTA, MAX, PDE4B, RAMP3, SDC2, TEK, TGFA, TPR | | 19 |
| cell stage | 0.032 | ACVR1, BAK1, BAP1, CCND1, FOXM1, IFNG, IL4, IL7, MAX, MCM10, MYB, NDC80, POLD1, PTOV1, SDC2, SOX10, TGFA, TPR | | 18 |
| survival of eukaryotic cells | 0.047 | CCND1, CD300A, CSF2RA, CXCL9, DUSP5, FDXR, IFNG, IL4, IL7, LTA, MYB, NT5C3, SOX10, TGFA, ZMYM2 | | 15 |
| activation of cells | 0.047 | ANK3, C4B, CCND1, CD300A, CXCL9, GNLY, IFNG, IL4, IL7, IL27, LTA, SDC2, TGFA, TOLLIP | | 14 |
| inflammatory response | 0.047 | CCND1, CSF2RA, CXCL9, EDNRB, GNLY, IFNG, IL4, IL7, LTA, PDE4B, PTGER2 | | 11 |
| morphology of cells | 0.047 | BAK1, CCND1, EDNRB, FOXM1, GNLY, IFNG, IL4, SDC2, TGFA, TPR | | 10 |
| proliferation of tumor cells | 0.011 | CSF2RA, FOXM1, IFNG, IL4, IL7, LTA, SDC2, TGFA | | 8 |
| **(A) 10 days post-SIV challenge** | | | | |
| **Function Annotation** | **B-H p-value** | **Molecules** | **# Molecules** | |
| tumorigenesis | 0.020 | ACVR1, AGPAT6, AKR1C3, ARAF, ARRB2, BARD1, BNIP3, BST2, BUB1, CD22, CSF2RA, EPHX1, EREG, FDFT1, GHRH, HIP1, IKBKE, IL10, IL12A, JUP, KDM1A, LIG1, MCM3, MET, NRG1, OPRM1, PLAUR, PPAP2A, PPPDE1, PRDX6, PRKCB, PSMB9, PTGS2, SDHD, SERPINA1, SERPING1, SLC25A5, SLC7A5, SPHK1, SPP1, TIMP1, TP53INP1, WT1, XPO5 | 44 | |
| inflammatory disorder | 0.020 | ACVR1, AMPD3, ARF1, ARRB2, C3AR1, CA4, CD22, CHCHD3, CLEC4D, CRLF2, CSF2RA, DENND1B, EREG, FBXO36, FCER1G, FDFT1, GRK6, HIP1, HIST1H2AC, HLA-DMB, IL10, IL12A, KIAA1267, NFASC, NRG1, OPRM1, P4HA2, PLAUR, PRDX6, PRKCB, PSMB9, PTGS2, SAMSN1, SERPINA1, SLC16A10, SPP1, TAP1, TIMP1, TKT, TP53INP1, UBAC2, USP3, WT1 | 43 | |
| cell death | 0.007 | ABCB7, ACVR1, ARRB2, BARD1, BCL2A1, BNIP3 (includes EG:664), BUB1, CD22, CSF2RA, CYB5R3, DFFA, EPHX1, FCER1G, FDFT1, GTF2F2, HIP1, HK1, IKBKE, IL10, IL12A, JUP, LIG1, LY86, LZTS2, MET, NRG1, OPRM1, PLAUR, PRDX6, PRKCB, PSMG2, PTGS2, RNF34, SERINC3, SERPINA1, SMARCA5, SPHK1, SPP1, TIMP1, TP53INP1, WT1 | 41 | |
| proliferation of cells | 0.009 | ABCB7, AKR1C3, ARAF, ATPIF1, BARD1, BCL2A1, BST2, BUB1, C3AR1, CD22, CSF2RA, EREG, FDFT1, GHRH, HK1, IKBKE, IL10, IL12A, JUP, KDM1A, LIG1, LY86, MET, NRG1, OPRM1, PLAUR, PRKCB, PTGS2, PTP4A3, SERINC3, SERPINA1, SLC7A5, SPHK1, SPP1, TIMP1, TP53INP1, WT1 | 37 | |
| growth of cells | 0.005 | ACTN1, ARAF, ARF1, ARRB2, BCL2A1, BNIP3, CD22, CSF2RA, EREG, FCER1G, GHRH, HIP1, HK1, IL10, IL12A, JUP, LZTS2, MCM3, MET, MGAT1, NRG1, PLAUR, PRKCB, PTGS2, PTP4A3, SERPINF2, SMARCA5, SPHK1, SPP1, TAP1, TIMP1, USP3, WT1 | 33 | |
| infectious disorder | 0.043 | ACTN1, ARF1, C3AR1, CA4, CD22, CSF2RA, FCER1G, FDFT1, HIST1H2AC, IKBKE, IL10, IL12A, KARS, LY86, MGAT1, OPRM1, PRDX6, PTGS2, SEC14L1, SERPINA1, SPP1, ST3GAL5, TIMP1, TKT | 24 | |
| developmental process of organism | 0.006 | ABCB7, ACVR1, BARD1, BST2, CSF2RA, DNMT3L, FDFT1, GHRH, HIP1, IL12A, KDM1A, MET, MGAT1, NRG1, PLAUR, PRKCB, PTGS2, SERPINA1, SMARCA5, SPHK1, TAP1, TIMP1, TKT | 23 | |
| migration of eukaryotic cells | 0.009 | ACVR1, ARRB2, CD22, CSF2RA, EREG, FCER1G, GRK6, IL10, IL12A, JUP, MET, NEUROG2, NRG1, OPRM1, PLAUR, PPAP2A, PRKCB, PTGS2, PTP4A3, SERPINA1, SPHK1, SPP1, TIMP1 | 23 | |
| cell division process | 0.025 | ACVR1, ARAF, BARD1, BCL2A1, BUB1, EREG, IL10, IL12A, KNTC1, LIG1, LZTS2, MET, NRG1, PLAUR, PPAP2A, PPP6C, PRKCB, PSMG2, PTGS2, SPHK1, SPP1, TP53INP1, WT1 | 23 | |
| development of cells | 0.043 | ACVR1, ARRB2, BCL2A1, CRLF2, CSF2RA, DFFA, FCER1G, IKBKE, IL10, IL12A, LIG1, MET, NEUROG2, NRG1, PLAUR, PSMB9, PTGS2, SMARCA5, SPHK1, SPP1, TM7SF4, WT1 | 22 | |
| Biological functions were determined for genes that had significantly different expression in the DNA+RANTES compared to both the DNA and control groups by Ingenuity Pathway Analysis. Analysis considered the 156 gene sequences at 8 months post-vaccination and the 159 gene sequences at 10 days post-SIV challenge as determined by Student’s post-hoc analysis of one-way ANOVAs at each time point. Functions were sorted according to the greatest number of molecules represented within the function. Biological functions listed were trimmed to reduce redundancy. Benjammini-Hochberg test correction was applied to P-value for biological function annotation. | | | | |
